# Exosomal circ_0050688 Shapes a Chemoresistant Microenvironment by Driving Spatial Resistance Spreading in Glioblastoma via the MDM2 Pathway

**DOI:** 10.3390/biom16060906

**Published:** 2026-06-18

**Authors:** Qiang Li, Jianglong Xu, Yuhao Zhang, Junbing Qian, Diana Bee-Lan Ong, Kein Seong Mun, Yiping Tang, Xiuchao Geng, Kean Chang Phang

**Affiliations:** 1 School of Medicine, Taizhou University, Taizhou 318000, Zhejiang, China; qiangli2020@tzc.edu.cn (Q.L.);; 2 Department of Pathology, Faculty of Medicine, Universiti Malaya, Kuala Lumpur 50603, Malaysia; 3 Department of Psychosomatic, Taizhou Second People’s Hospital, Affiliated to School of Medicine, Taizhou University, Taizhou 318000, Zhejiang, China; 4 Department of Neurosurgery, Affiliated Hospital of Hebei University, Baoding 071000, Hebei, China; 5 Department of Neurosurgery, Zhejiang Provincial People’s Hospital, Affiliated to Hangzhou Medical College, Hangzhou 310014, Zhejiang, China

**Keywords:** glioblastoma, exosomes, circ_0050688, acquired chemoresistance, MDM2

## Abstract

**Background:** Acquired tolerance to temozolomide (TMZ) remains one of the main obstacles to enduring therapeutic success in glioblastoma (GBM). While tumor-derived extracellular vesicles are known to orchestrate therapy evasion by horizontally transferring molecules across the tumor microenvironment, the precise regulatory roles of specific exosomal circular RNAs (circRNAs) in establishing this refractory state require further elucidation. **Methods:** The expression of circ_0050688 in TMZ-resistant GBM clinical tissues and cell lines was evaluated. Exosomes derived from resistant cells were isolated and confirmed via transmission electron microscopy (TEM) and marker analysis. PKH67 fluorescent tracking was utilized to visually demonstrate exosome internalization by sensitive recipient cells. Biological functions, including the expression of the multidrug resistance protein P-glycoprotein (P-gp) and the proliferation marker Ki-67, were evaluated. The competing endogenous RNA mechanism was validated using RNA FISH, dual-luciferase reporters, and functional rescue experiments. In vivo efficacy was determined using subcutaneous xenograft mouse models. **Results:** Clinical and in vitro analyses revealed that circ_0050688 is upregulated in TMZ-refractory GBM, predicting adverse patient survival. Through PKH67-based tracing, we confirmed that resistant cells actively secrete circ_0050688-enriched exosomes, which are subsequently engulfed by drug-sensitive bystander cells. This vesicular transfer directly instigates a chemoresistant and highly proliferative phenotype, marked by elevated P-gp and Ki-67 levels. At the molecular level, circ_0050688 operates as a molecular decoy for miR-508-5p, thereby preventing the suppression of its downstream target, MDM2. Functionally, circ_0050688 depletion eradicated these aggressive traits and restored TMZ vulnerability across both cellular and murine xenograft models. Furthermore, rescue assays confirmed that this circ_0050688-driven chemoresistance is fundamentally dependent on the miR-508-5p/MDM2 signaling axis. **Conclusions:** Current data uncover an intercellular signaling network driven by vesicular circ_0050688, which functions as a mobile oncogene to reshape the TMZ-refractory microenvironment. Targeting this exosomal circ_0050688/miR-508-5p/MDM2 network to suppress P-gp and Ki-67 expression represents a highly promising therapeutic strategy for refractory GBM.

## 1. Introduction

As the most frequently diagnosed and lethal malignant primary brain tumor, glioblastoma (GBM) is notoriously defined by its extreme cellular heterogeneity and invariably fatal disease course [1,2]. Despite rigorous therapeutic interventions combining tumor resection with concurrent chemoradiation, patient prognosis is severely constrained, yielding a median survival of only 12–15 months [3,4]. Temozolomide (TMZ) remains the cornerstone of first-line chemotherapy for GBM due to its exceptional ability to penetrate the blood–brain barrier (BBB) [5]. However, its long-term therapeutic efficacy is ultimately defeated by the inevitable emergence of acquired chemoresistance during prolonged treatment [6,7]. Indeed, alongside other formidable challenges, this acquired TMZ resistance remains one of the main obstacles to enduring therapeutic success, frequently driving fatal tumor recurrence and rendering subsequent interventions largely ineffectual [8]. Consequently, deciphering the biological basis of TMZ refractoriness and exploring highly efficient, BBB-penetrable drug delivery systems are urgent clinical imperatives.

Circular RNAs (circRNAs) are covalently closed transcripts that critically modulate epigenetic networks, thereby promoting tumor malignancy and therapeutic resistance [9,10,11]. Owing to their exceptional structural stability, circRNAs are highly enriched in exosomes as tumor-derived extracellular nanovesicles [12,13]. Exosomes function as crucial intercellular communication vehicles within the tumor microenvironment (TME). Importantly, they possess the intrinsic capacity to penetrate the BBB and release functional cargo, including circRNAs, to neighboring or distant recipient cells, thereby spatially propagating malignant phenotypes and chemoresistance across the tumor bulk [14,15,16]. While circRNAs frequently function as competing endogenous RNAs (ceRNAs) to sequester microRNAs (miRNAs) and derepress downstream oncogenes [17,18,19], the specific exosomal circRNA networks orchestrating the intercellular transmission of TMZ resistance in GBM remain largely unexplored.

Previous studies have implicated the dysregulation of specific miRNAs, such as the tumor-suppressive miR-508-5p, in glioma pathogenesis [20]. However, the upstream post-transcriptional mechanisms silencing miR-508-5p and its precise downstream effectors in the context of acquired TMZ resistance are yet to be defined. Murine double minute-2 (MDM2), a canonical E3 ubiquitin ligase, is a potent oncogene that drives tumor progression and chemoresistance. Whether an exosome-mediated ceRNA network governs the miR-508-5p/MDM2 axis to dictate GBM therapeutic adaptation remains a critical open question.

Here, we identify circ_0050688 as a novel oncogenic driver and a central factor promoting acquired resistance to TMZ in GBM. We demonstrate that circ_0050688 is dramatically enriched in exosomes derived from TMZ-resistant cells and can be intercellularly transferred to sensitive recipient cells, effectively conferring chemoresistance. Mechanistically, exosomal circ_0050688 competitively sequesters miR-508-5p to augment MDM2, thereby driving malignant progression and therapeutic failure. By elucidating the exosomal circ_0050688/miR-508-5p/MDM2 signaling axis, our findings characterize a basic pathway for spatial resistance propagation in the TME and offer persuasive justification for interfering with this network to overcome TMZ-resistant recurrent GBM.

## 2. Materials and Methods

### 2.1. Clinical Specimens and Ethical Approval

A total of 45 patients with primary GBM receiving surgical intervention at the Affiliated Hospital of Hebei University from 2022 to 2025 were recruited. Crucially, to strictly adhere to the 2021 WHO Classification of Tumors of the Central Nervous System, all enrolled tumor specimens were subjected to IDH1 R132H immunohistochemistry (IHC) screening, and only tumors definitively confirmed as IDH-wildtype were included in this cohort. Furthermore, MGMT promoter methylation status was clinically assessed using an NGS-based brain tumor 460-gene panel (Genecast Biotechnology, Beijing, China) and was included as a key covariate for subsequent prognostic calibration.

All patients underwent the standard Stupp protocol strictly in accordance with NCCN guidelines, consisting of maximal safe resection followed by concurrent chemoradiotherapy (total dose of 60 Gy; daily oral TMZ at 75 mg/m^2^) and subsequent adjuvant TMZ maintenance (150–200 mg/m^2^ for 6 cycles). Routine blood monitoring was performed; while mild hematological toxicities were managed symptomatically, any patient who required early termination of TMZ therapy due to severe leukopenia or thrombocytopenia was strictly excluded from the final cohort to avoid confounding the resistance stratification.

The clinical cohort comprised 20 TMZ-resistant and 25 TMZ-sensitive patients. All GBM diagnoses were definitively confirmed via histopathological examination. Clinically, TMZ resistance was defined as documented disease progression during the standard adjuvant chemotherapy phase or tumor recurrence within six months post-treatment. Conversely, TMZ sensitivity was defined as the absence of recurrence or disease relapse occurring more than six months after the completion of standard chemotherapy regimen. Upon surgical extraction, all excised tumor tissues were instantly frozen in liquid nitrogen and preserved until further molecular analysis. The study protocol was formally approved by the Medical Ethics Committee of the Affiliated Hospital of Hebei University (Approval No. HDFY-LL-2022-067). Before enrollment, we secured signed informed consent from all patients or their legally authorized representatives. All procedures involving human participants were conducted in strict adherence to the ethical principles articulated in the Declaration of Helsinki by the World Medical Association.

### 2.2. Cell Culture and Authentication

U251 MG and U343 MG GBM cells were commercially procured from GuangZhou Jennio Biotech (ExPASy: CVCL_0021 and CVCL_0580, respectively, Guangzhou, China). To rigorously verify their genetic fidelity and unequivocally rule out cross-contamination, independent short tandem repeat (STR) profiling was executed prior to all functional assays by a third-party facility (Genetic Testing Biotechnology Corp., Suzhou, China).

### 2.3. Establishment of TMZ-Resistant Lines

Standard culture conditions comprised a 37 °C, 5% CO_2_ environment using DMEM (Thermo Fisher Scientific, Waltham, MA, USA) enriched with 10% FBS (HyClone, Logan, UT, USA). The acquired chemoresistance models, U343-R and U251-R, were generated following our recently validated dose-escalation protocol [21,22,23]. Instead of a continuous bulk description, parental cohorts underwent a 6-month selection phase utilizing incremental doses of temozolomide (Sigma-Aldrich, St. Louis, MO, USA, reconstituted in DMSO). Each progressive TMZ concentration was maintained for roughly 20 days until the refractory phenotype stabilized, which was subsequently verified by CCK-8 viability screening. To sustainably preserve this resistance trait, sublines were challenged weekly with a 100 µM TMZ maintenance dose for 24 h.

### 2.4. circRNA Microarray Profiling and Bioinformatic Analysis

To identify differentially expressed circRNAs, exosomes were first isolated from the TMZ-resistant GBM cells. Total exosomal RNA was subsequently extracted and purified utilizing the Qiagen Serum/Plasma Kit (Qiagen, Hilden, Germany). The circRNA expression profiling was performed by Shanghai Biotechnology Corporation using the Agilent Human circRNA Microarray platform. For transcriptomic data processing, raw microarray data were subjected to quantile normalization and background correction utilizing the limma package within the R statistical computing environment. Differentially expressed circRNAs were defined by an absolute fold change ≥ 2.0 and a *p*-value < 0.05. Finally, hierarchical clustering and heatmap visualization of the significantly altered circRNAs were conducted using the Hiplot platform [24].

### 2.5. Bioinformatics Analysis and ceRNA Network Construction

Genomic characteristics of circ_0050688 were retrieved from the circBase and circBank databases. Potential microRNA (miRNA) targets of circ_0050688 were predicted using circBank, circMine. Subsequently, potential miR-508-5p targets were screened via the TargetScan, circNET, miRTarBase, miRDB, miRWalk, and miRPathDB platforms. Overlapping candidate genes across these databases were identified as the final targets. We employed miRanda and RNAhybrid to map the binding sequences between circ_0050688 and miR-508-5p, and subsequently determined the circRNA’s secondary structure via RNAfold.

### 2.6. Sanger Sequencing and Back-Splicing Junction (BSJ) Validation

To unequivocally verify the circular characteristics and the precise BSJ of circ_0050688, cDNA was synthesized from total RNA isolated from GBM cells through reverse transcription. Subsequently, the target region encompassing the BSJ was amplified via PCR utilizing specifically designed divergent primers. The resulting PCR amplicons were purified and subjected to bidirectional Sanger sequencing by Tsingke Biotechnology (Beijing, China). Finally, the acquired sequencing chromatograms were aligned and mapped against the reference sequences documented in the circBase database to definitively confirm the back-splicing site.

### 2.7. RNase R Digestion Assay

The circularity and stability of the transcripts were confirmed by digesting total RNA with RNase R (3 U/μg; Epicentre, Madison, WI, USA) at 37 °C (30 min), alongside a mock-treated control [23]. The RNeasy MinElute Cleanup Kit (Qiagen) was used to purify the digested RNA ahead of RT-qPCR.

### 2.8. Transcription Inhibition and RNA Half-Life Assay

To evaluate the transcript stability and determine the intracellular half-life of circ_0050688, cultured GBM cells were exposed to 2 μg/mL Actinomycin D (Sigma-Aldrich, St. Louis, MO, USA) to inhibit new RNA transcription. After blocking transcription, cells were collected at 4 h increments up to 12 h, followed by extraction of cellular RNA. Subsequently, the residual expression levels of circ_0050688, alongside its cognate linear mRNA counterpart, were quantified via RT-qPCR. The RNA decay kinetics and corresponding half-life values were mathematically modeled and statistically analyzed utilizing GraphPad Prism software.

### 2.9. RNA Extraction and Quantitative Real-Time PCR (RT-qPCR)

Total RNA was isolated from cultured GBM cells, xenograft tissues, and purified exosomes utilizing TRIzol Reagent (Takara, Kyoto, Japan), followed by spectrophotometric quantification of RNA concentration and integrity. For mRNA and circ_0050688, we utilized the iScript cDNA Synthesis Kit (Bio-Rad, Hercules, CA, USA) to generate cDNA. Conversely, mature miR-508-5p, total RNA was reverse-transcribed using the Mir-X™ miRNA First-Strand Synthesis Kit (Takara, Kyoto, Japan). RT-qPCR was subsequently on an ABI 7500 Real-Time PCR System (Applied Biosystems, Foster City, CA, USA) using the SYBR Premix Ex Taq kit (Takara). Relative expression was calculated via the 2^−ΔΔCt^ method, with data normalized to GAPDH (for mRNA/circ_0050688) or U6 (for miR-508-5p). A complete list of primer sequences is available in Supplementary Table S1.

### 2.10. Fluorescence In Situ Hybridization (FISH)

To assess the subcellular positioning of circ_0050688, FISH was conducted. Cells were plated in 48-well formats at a density of 1 × 10^4^ cells per well. Following fixation with 4% paraformaldehyde (PFA; Biosharp, Anhui, China), cell permeabilization was carried out using 0.5% Triton X-100. A specific FITC-labeled oligonucleotide probe targeting the unique BSJ of circ_0050688, was synthesized via GenePharma (Shanghai, China). The hybridization procedure was executed utilizing a commercial FISH Kit (GenePharma) adherence to the manufacturer’s protocols. Following nuclear labeling with DAPI, high-resolution confocal micrographs were captured using a Zeiss LSM980 microscope (Carl Zeiss, Oberkochen, Germany).

### 2.11. Dual-Luciferase Reporter Assay

To validate the binding of miR-508-5p to circ_0050688 and MDM2, the respective WT and MUT target segments were integrated into pmirGLO plasmids (Promega, Madison, WI, USA). TMZ-resistant GBM cells were co-transfected with these engineered constructs alongside miR-508-5p or NC mimics. Forty-eight hours post-transfection, luminescence was quantified via Promega’s dual-luciferase detection kit, determining the ratio of Firefly to Renilla signals.

### 2.12. Cell Transfection and Lentiviral Transduction

GeneChem (Shanghai, China) engineered the lentiviral vectors expressing the circ_0050688-targeting and control shRNAs. Additionally, we procured all miR-508-5p modulators (mimics and inhibitors) alongside their matching controls from Thermo Fisher Scientific (Waltham, MA, USA). The MDM2 overexpression plasmid and the control empty vector were obtained from Addgene (Cambridge, MA, USA). Cells were transiently transfected with miRNA oligonucleotides or MDM2 plasmids using Lipofectamine 3000 (Thermo Fisher Scientific). To establish stable knockdown lines, cells were transduced with lentiviruses (with 8 μg/mL polybrene) and selected with puromycin 48 h post-infection. Supplementary Table S2 lists all oligonucleotide sequences used.

### 2.13. EdU Incorporation Assay

Cellular proliferation was evaluated utilizing the Cell-Light EdU Apollo In Vitro Kit (RiboBio) according to our previously described methodology [23]. Following incubation with 10 μM EdU for 2 h, the cells underwent fixation and Apollo reaction staining. Proliferation rates were calculated as the percentage of EdU-labeled cells relative to the total Hoechst 33342-stained nuclei, visualized under an Olympus fluorescence microscope.

### 2.14. In Vitro Chemosensitivity and Half-Maximal Inhibitory Concentration (IC_50_) Determination

TMZ (0–1600 μM) was applied to GBM cells cultured in 96-well plates for 48 h. The Cell Counting Kit-8 (CCK-8; Beyotime Biotechnology, Shanghai, China) was employed to determine cell viability after a 4 h incubation period. Absorbance at 450 nm was measured using a TriStar LB941 microplate reader (Berthold Technologies, Bad Wildbad, Germany). IC_50_ values were calculated via non-linear regression analysis.

### 2.15. Cell Migration and Invasion Assays

Cell migration and invasion were assessed using 8.0 µm Transwell inserts (Corning, NY, USA), uncoated or pre-coated with Matrigel (BD Biosciences, San Jose, CA, USA), respectively. After 48 h, non-migrating cells on the upper surface were removed with a cotton swab. Migrated or invaded cells that successfully crossed to the inferior membrane were immobilized in 4% PFA (30 min), subjected to coloring with 0.1% crystal violet (15 min), and enumerated in arbitrary fields via microscopic examination.

### 2.16. Protein Extraction and Western Blot Analysis

Whole-cell lysates were harvested using RIPA lysis buffer (KeyGEN Biotech, Nanjing, China) containing a protease inhibitor cocktail. Equivalent protein loads were resolved via SDS-PAGE and electroblotted onto 0.45-μm polyvinylidene difluoride (PVDF) membranes (Millipore). Non-specific binding was minimized by a 1 h blocking step with 5% skim milk. Subsequently, membranes were probed overnight at 4 °C using antibodies specific for MDM2 (1:500, AF6376; Affinity), GST-π (1:1000, 15902-1-AP; Proteintech), MGMT (1:1000, #2739; CST), and P-gp (1:1000, ab170904; Abcam), with GAPDH (1:3000, AF7021; Affinity) or β-actin (1:5000, 66009-1-Ig; Proteintech) as loading controls. After thorough washing, the membranes underwent a 1 h incubation with HRP-labeled secondary antibodies under ambient conditions. Where necessary, blots were stripped and re-probed. Bands were visualized using an ECL substrate on a Tanon 6600 workstation and quantified with Image-Pro Plus 6.0, with signals normalized to GAPDH or β-actin.

### 2.17. Exosome Isolation and Characterization

Exosomes were harvested from conditioned media (enriched with vesicle-free FBS) through a series of ultracentrifugation steps, as we previously established [21,25], strictly adhering to the MISEV2018 guidelines [26]. To isolate extracellular vesicles, the collected conditioned media underwent a series of clearance spins at 4 °C to eliminate intact cells, dead cells, and larger debris. The resulting supernatant was passed through a 0.22-μm Millipore filter. Exosomes were subsequently pelleted from the clarified liquid via ultracentrifugation (110,000× *g*, 90 min, 4 °C) via a Hitachi CP100MX system. For optimal vesicle purity, the precipitates underwent PBS rinsing followed by a repeat ultracentrifugation cycle and ultimately resuspended in PBS. For morphological evaluation, the exosome was visualized utilizing a transmission electron microscope (TEM; HT-7700, Hitachi, Japan) at 100 kV. Size distribution was profiled utilizing a high-resolution Flow NanoAnalyzer (NanoFCM Inc., Xiamen, China). Exosome markers (CD63, TSG101) and the negative control (Calnexin) were validated by Western blotting.

### 2.18. Transwell Co-Culture and Exosome Inhibition Assay

To evaluate the exosome-mediated intercellular transfer of circ_0050688, TMZ-sensitive recipient cells were seeded in the lower chambers of 6-well 0.4 μm Transwell systems (Corning, NY, USA), while TMZ-resistant cells were plated in the upper inserts. To inhibit exosome release, U343-R cells were treated with 10 μM GW4869 (Sigma-Aldrich, St. Louis, MO, USA) or DMSO (vehicle control). Recipient cells were collected at 48 h post-co-culture, and their internal circ_0050688 levels were subsequently measured via RT-qPCR.

### 2.19. Exosome Labeling and Cellular Uptake Assay

Purified exosomes were fluorescently labeled utilizing the PKH67 Green Fluorescent Cell Linker Kit (Sigma-Aldrich, St. Louis, MO, USA) in accordance with the manufacturer’s instructions. After a 5 min incubation, the reaction was quenched with 1% bovine serum albumin. Excess dye was removed by ultracentrifugation (100,000× *g*, 70 min, 4 °C) and PBS washing. To evaluate cellular uptake, recipient U343 cells were exposed to the PKH67-tagged vesicles. Twenty-four hours post-incubation, the samples underwent 4% PFA fixation and DAPI nuclear counterstaining prior to confocal microscopic imaging.

### 2.20. Immunofluorescence Staining and Confocal Microscopy

Immunofluorescence assays were conducted following our previously established protocols [23]. Once fixed and permeabilized, the GBM cells were labeled overnight using primary antibodies directed toward Ki-67 (1:400; Cell Signaling Technology) and P-gp (1:100; Thermo Fisher Scientific). Visualization was achieved via Alexa Fluor 488-conjugated antibodies and DAPI counterstaining. Confocal microscopy was used to obtain fluorescence signals, followed by quantification through ImageJ 1.54g software (National Institutes of Health, Bethesda, MD, USA).

### 2.21. Subcutaneous Tumor Xenograft Models

All animal procedures were strictly conducted in accordance with the institutional animal welfare guidelines and were approved by the Animal Ethics and Welfare Committee of Taizhou University (Approval No.: TZXY-2024-20241158). Male BALB/c nude mice (5–6 weeks old) were purchased from Changzhou Caven Laboratory Animal Co., Ltd. (Changzhou, China). To establish the xenograft models, mice were inoculated subcutaneously into the right axilla with 0.2 mL of a cell suspension containing 1 × 10^7^ cells (prepared at a concentration of 5 × 10^7^ cells/mL). Tumor dimensions were monitored using a digital caliper every 3 days, and tumor volumes were calculated utilizing the formula: Volume = 0.5 × length × width^2^ [27]. Once tumor volumes reached approximately 100 mm^3^, mice were randomly assigned to treatment groups (*n* = 6).

For the chemosensitization model, mice were inoculated with U343-R cells (shRNA-NC or shRNA-circ_0050688). Upon tumor formation, mice received intraperitoneal (i.p.) injections of TMZ (20 mg/kg in 0.8% DMSO) or vehicle every 3 days for 9 doses. The TMZ dosage and regimen were determined based on our previous study [21] and established protocols [28,29]. To evaluate exosome transfer, mice harboring parental U343 tumors were intratumorally (i.t.) injected with exosomes (60 μg protein in 30 μL PBS) [30,31,32] or PBS vehicle every 3 days for 9 doses. Body weights were recorded every 3 days to monitor toxicity. After 28 days of treatment, mice were euthanized via CO_2_ asphyxiation. Tumor tissues were harvested, documented photographically, and measured for weight prior to processing for downstream molecular and Western blot analyses.

### 2.22. Statistical Analysis

All quantitative values represent the mean ± standard deviation (SD). In vitro cell experiments were performed with triplicate independent biological replicates (*n* = 3 per group), while in vivo animal experiments contained six animals per experimental group (*n* = 6 per group). Statistical evaluations and data visualization were performed using GraphPad Prism software (version 10.1.2). Significance between two independent groups was assessed via an unpaired, two-tailed Student’s *t*-test. Conversely, multi-group comparisons utilized one- or two-way ANOVA coupled with Tukey’s or Sidak’s *post hoc* procedures for multiple testing corrections. When appropriate, Kaplan–Meier survival curves were generated, and differences between survival curves were evaluated using the log-rank test. Statistical significance was accepted at *p* < 0.05, and identified with “*”.

### 2.23. AI-Assisted Language Improvement

During the preparation of this manuscript, the authors used DeepSeek (version DeepSeek-V3, Hangzhou, China) solely for the purpose of English language editing, grammatical correction, spell-checking, and overall readability improvement. Following the use of this tool, all authors rigorously reviewed and edited the modified content, as necessary. The authors assume full responsibility for the final content and integrity of this publication.

## 3. Results

### 3.1. circ_0050688 Is a Stable, Cytoplasm-Enriched Transcript Upregulated in TMZ-Resistant GBM

**Figure 1:**
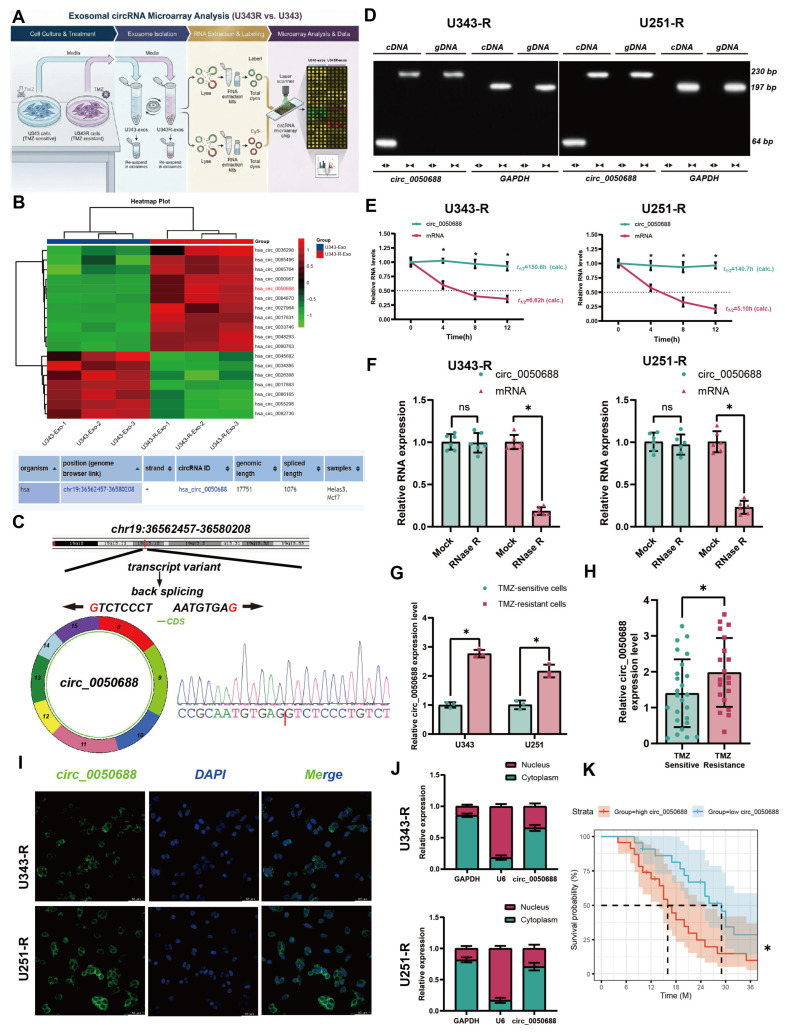
circ_0050688 is a stable circular RNA upregulated in TMZ-resistant GBM and predicts poor clinical prognosis. (**A**) Schematic illustration of the exosomal circRNA microarray analysis workflow comparing TMZ-resistant (U343-R) and TMZ-sensitive (U343) cells. (**B**) **Upper panel**: Heatmap profiling the top differentially expressed exosomal circRNAs, with circ_0050688 highlighted in red. **Lower panel**: Basic genomic characteristics of circ_0050688 based on circBase. (**C**) **Main schematic**: Diagram illustrating the genomic locus and back-splicing formation of circ_0050688. **Lower-right inset**: Sanger sequencing chromatogram validating the back-splice junction site. (**D**) Agarose gel electrophoresis of PCR products amplified from cDNA and gDNA using divergent and convergent primers. GAPDH served as a linear control. (**E**) Evaluation of circ_0050688 and its linear host mRNA stability in U343-R and U251-R cells following Actinomycin D treatment, * vs. linear mRNA at the corresponding time points. (**F**) Relative RNA expression levels of circ_0050688 and the linear host mRNA after RNase R exonuclease digestion, assessed via qRT-PCR, * vs. Mock group (without RNase R treatment); not significant (n.s.). (**G**) qRT-PCR analysis of circ_0050688 expression levels in TMZ-sensitive (U343, U251) and TMZ-resistant (U343-R, U251-R) GBM cell lines, * vs. U343 group; * vs. U251 group. (**H**) Relative expression of circ_0050688 in clinical GBM tissue specimens (*n* = 25 for TMZ-sensitive, *n* = 20 for TMZ-resistant), evaluated by qRT-PCR. * vs. TMZ-sensitive group. (**I**) Representative FISH images demonstrating the subcellular localization of circ_0050688 (green) in U343-R and U251-R cells. Nuclei were counterstained with DAPI (blue). Scale bar = 50 μm. (**J**) Subcellular fractionation followed by qRT-PCR to quantify the nucleocytoplasmic distribution of circ_0050688. GAPDH and U6 were utilized as cytoplasmic and nuclear internal controls, respectively. (**K**) Kaplan–Meier survival curve illustrating the correlation between circ_0050688 expression levels and the overall survival of GBM patients. Patients were stratified into high- and low-expression groups based on the median expression value, * vs. low- circ_0050688 group.

To clarify how circRNA cargo within exosomes orchestrates the refractory response to TMZ in GBM, we initially performed a circRNA microarray analysis comparing exosomes derived from TMZ-resistant and TMZ-sensitive cells (Figure 1A). Differential expression profiling revealed a distinct circRNA signature, with hsa_circ_0050688 standing out as a prominently enriched transcript in the TMZ-resistant group (Figure 1B). Genomic annotation from circBase indicates that circ_0050688 is located on chromosome 19. We first verified the circular nature of circ_0050688. Sanger sequencing confirmed the exact sequence spanning the unique BSJ (Figure 1C). Furthermore, PCR amplification coupled with agarose gel electrophoresis demonstrated that divergent primers successfully amplified circ_0050688 exclusively from cDNA, but not from genomic DNA (gDNA), confirming its circular configuration (Figure 1D). Actinomycin D assays confirmed the superior stability of circ_0050688, which exhibited a significantly longer half-life than its linear counterpart (Figure 1E). Similarly, circ_0050688 was highly resistant to RNase R digestion, whereas the linear host mRNA was readily degraded (Figure 1F), confirming its circular structure.

Subsequently, the expression patterns and clinical relevance of circ_0050688 were examined. Validation via qRT-PCR revealed a notable enrichment of circ_0050688 in TMZ-refractory GBM models in contrast with their drug-sensitive counterparts (Figure 1G). In our clinical cohort, circ_0050688 was upregulated in the TMZ-resistant group (Figure 1H), confirming our initial microarray findings. Importantly, prognostic assessment using Kaplan–Meier curves revealed that elevated circ_0050688 levels are strongly associated with dismal clinical outcomes in the GBM cohort (*p* < 0.05) (Figure 1K). Furthermore, multivariate Cox regression analysis confirmed that high circ_0050688 expression remained an independent poor prognostic factor for these patients (HR = 1.35, 95% CI = 1.01–1.81, *p* = 0.042; Supplementary Figure S1). In contrast, MGMT promoter unmethylation (HR = 1.54, 95% CI = 0.85–2.79, *p* = 0.156) and age ≥ 60 years (HR = 1.12, 95% CI = 0.59–2.13, *p* = 0.724) lacked statistical significance and exhibited no trend toward differences.

Both FISH (Figure 1I) and nucleocytoplasmic fractionation (Figure 1J) revealed that circ_0050688 is predominantly localized to the cytoplasm in U343-R and U251-R cells, suggesting its potential role as a ceRNA sponge. Collectively, these findings identify circ_0050688 as a highly stable, clinically upregulated circRNA implicated in GBM TMZ resistance.

### 3.2. circ_0050688 Depletion Sensitizes GBM Cells to TMZ and Inhibits Pro-Tumorigenic Traits In Vitro

**Figure 2:**
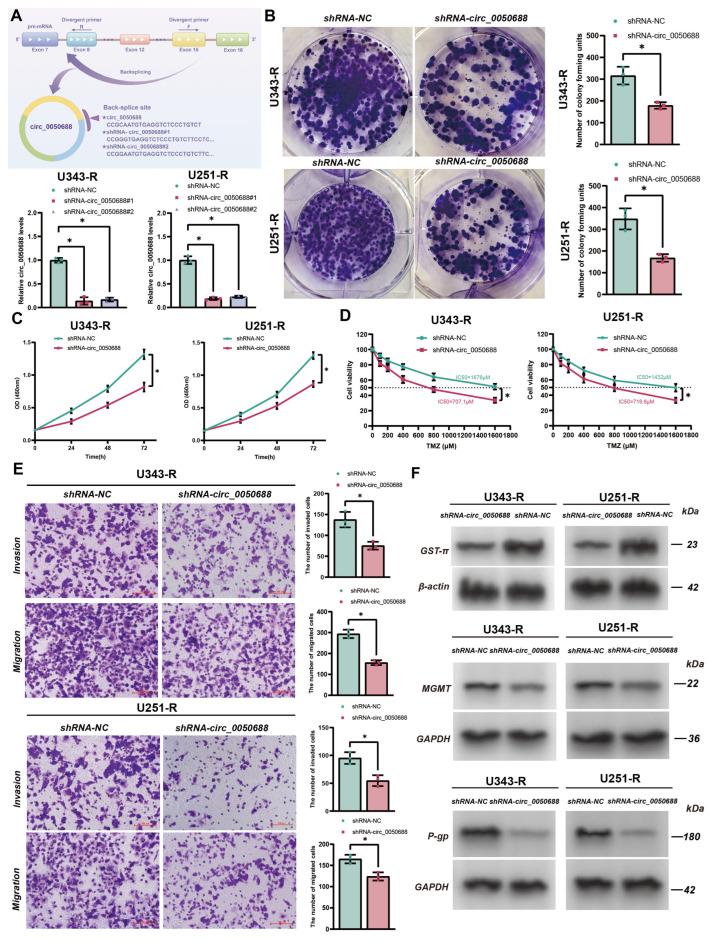
Silencing of circ_0050688 suppresses malignant phenotypes and reverses TMZ resistance in GBM cells. (**A**) **Upper panel**: Schematic representation of the shRNA design targeting the unique back-splice junction of circ_0050688. **Lower panel**: qRT-PCR validation of knockdown efficiency following transfection with two independent shRNAs (shRNA-circ_0050688#1 and shRNA-circ_0050688#2) versus a negative control (shRNA-NC) in U343-R and U251-R cell lines. (**B**) Representative images (**left**) and quantification (**right**) of colony formation in U343-R and U251-R cells following circ_0050688 knockdown. (**C**) CCK-8 proliferation curves of U343-R and U251-R cells following circ_0050688 knockdown. (**D**) Cell viability curves and IC_50_ values of TMZ-resistant cells treated with gradient TMZ concentrations following circ_0050688 knockdown. (**E**) Representative images and quantification of migration and invasion in U343-R (**upper**) and U251-R (**lower**) cells following circ_0050688 knockdown. Scale bars = 100 μm. (**F**) Representative Western blot images evaluating the protein expression levels of chemoresistance markers (GST-π, MGMT, and P-gp) following circ_0050688 knockdown. β-actin and GAPDH were used as loading controls. Panel (**A**) * vs. shRNA-NC group for both shRNA-circ_0050688#1 and shRNA-circ_0050688#2. Panels (**B**–**E**) * vs. shRNA-NC group.

Given the pronounced upregulation of circ_0050688 in TMZ-resistant GBM cells, we performed loss-of-function assays to elucidate its biological role. Two independent short hairpin RNAs (shRNA-circ_0050688#1 and #2) specifically targeting the unique BSJ were designed and synthesized. qRT-PCR analysis confirmed that both shRNAs achieved profound knockdown efficiency. shRNA-circ_0050688#1 (hereafter referred to as shRNA-circ_0050688) was selected for subsequent functional experiments (Figure 2A).

Functional evaluations demonstrated that circ_0050688 silencing markedly suppressed the proliferative and colony-forming potential of GBM cells (Figure 2B) and the proliferation (Figure 2C) of both TMZ-resistant cell lines. Silencing circ_0050688 significantly reduced the IC_50_ values of TMZ in U343-R/U251-R cells, effectively re-sensitizing them to TMZ (Figure 2D). Furthermore, circ_0050688 knockdown significantly impaired the migration and invasion of both resistant cell lines (Figure 2E).

To uncover the molecular basis underpinning this phenotypic sensitization, the expression profiles of classic chemoresistance-associated proteins were assessed. Western blot analysis demonstrated that circ_0050688 knockdown elicited a marked downregulation of key chemoresistance-associated proteins, including GST-π, MGMT, and P-gp, in TMZ-resistant cells (Figure 2F). Collectively, these in vitro findings definitively establish circ_0050688 as a critical oncogenic driver that sustains proliferation, invasion, and TMZ resistance in GBM.

### 3.3. Exosomal circ_0050688 Disseminates TMZ Resistance and Malignant Phenotypes via Intercellular Communication

**Figure 3:**
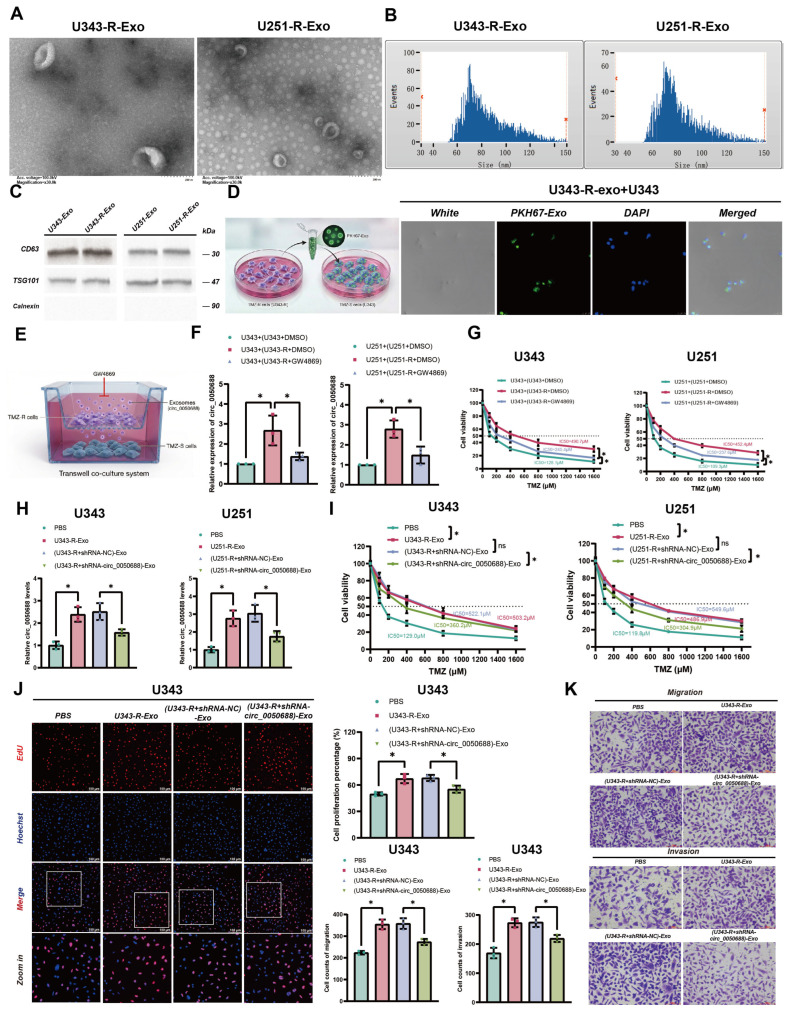
Exosome-mediated transfer of circ_0050688 disseminates TMZ resistance and promotes malignant phenotypes in vitro. (**A**) Representative TEM images showing the typical morphology of exosomes derived from U343-R and U251-R cells. (**B**) Size distribution of the purified exosomes measured by flow cytometry-based NanoFCM. (**C**) Western blot analysis of exosome markers (CD63, TSG101) and the negative marker (Calnexin) in the isolated exosomes. (**D**) **Left**: Schematic illustration of the exosome co-incubation assay. **Right**: Confocal microscopy images demonstrating the internalization of PKH67-labeled U343-R-Exo (green) into recipient U343 cells. Nuclei were stained with DAPI (blue). (**E**) Schematic representation of the Transwell co-culture system utilized to assess exosome-mediated intercellular communication in the presence or absence of the exosome secretion inhibitor GW4869. (**F**) qRT-PCR analysis of circ_0050688 expression in sensitive cells co-cultured with resistant cells in the presence or absence of GW4869. (**G**) Cell viability curves and calculated IC_50_ values of recipient sensitive cells co-cultured with resistant cells, indicating the reversal of acquired TMZ resistance upon GW4869 treatment. (**H**) Relative circ_0050688 levels in recipient cells treated with PBS or the indicated exosomes. (**I**) CCK-8 assays evaluating the TMZ sensitivity (IC_50_) of recipient cells following incubation with the indicated exosomes. (**J**) Representative images and quantitative analysis of EdU incorporation assays assessing the proliferation of U343 cells treated with the indicated exosomes. (**K**) Transwell assays evaluating the migratory and invasive capacities of U343 cells following treatment with the indicated exosomes. Panels (**F**,**G**) * vs. sensitive cells co-cultured with DMSO-treated sensitive cells; * vs. sensitive cells co-cultured with DMSO-treated resistant cells. Panels (**H**–**K**) * vs. PBS group; * vs. (resistant cells + shRNA-NC)-derived exosomes group.

Given that exosomes act as vital conduits for intercellular communication and chemoresistance dissemination [33,34,35], we hypothesized that exosomes serve as vehicles for circ_0050688, mediating its transmission from the resistant population to sensitive counterparts. We first isolated and characterized exosomes. The ultrastructure of the isolated vesicles, characterized by a representative cup-shaped morphology, was visualized via TEM (Figure 3A), and flow cytometry-based nanoparticle analysis (NanoFCM) confirmed a size distribution peaking at approximately 60–120 nm (Figure 3B). Moreover, the abundance of typical exosomal markers (CD63, TSG101) alongside the exclusion of calnexin were validated through Western blotting (Figure 3C), validating the successful isolation of exosomes.

To visually verify exosome internalization, PKH67-labeled U343-R-Exo was incubated with sensitive U343 cells. Confocal microscopy demonstrated striking internalization of green, fluorescent exosomes into the cytoplasm of target cells (Figure 3D). To determine whether circ_0050688 is transmitted via these vesicles, we established a Transwell co-culture system (Figure 3E). Co-culturing sensitive cells with their resistant counterparts significantly upregulated circ_0050688 expression in the sensitive recipient cells. We employed 10 μM GW4869 to specifically block nSMase2-mediated exosome release [36,37,38]. This optimal dosage aligns with standard glioma paradigms, ensuring that the observed transfer impairment resulted from targeted biogenesis inhibition rather than general cellular toxicity [36,39]. Crucially, the co-culture-induced upregulation of circ_0050688 was largely abrogated by the addition of GW4869 (Figure 3F). Consistently, GW4869 treatment profoundly reversed the acquired TMZ resistance (IC_50_ elevation) in sensitive cells (Figure 3G).

**Figure 4:**
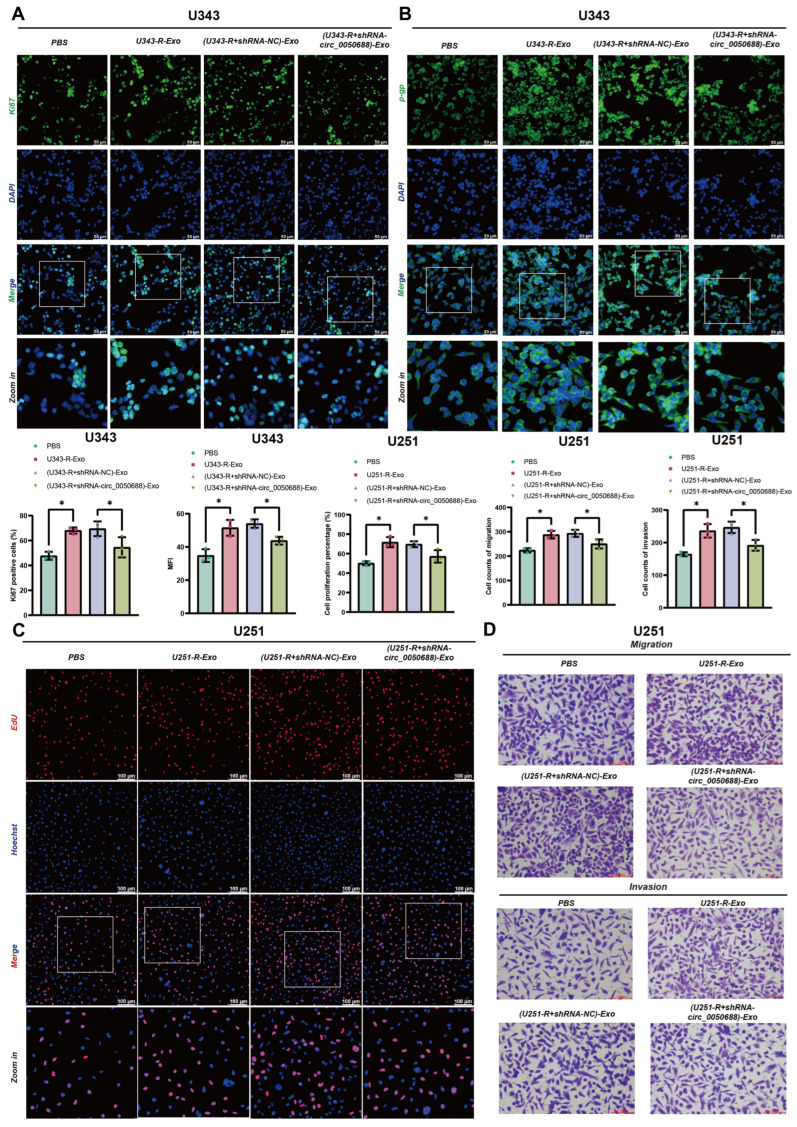
Exosomal circ_0050688 enhances proliferation, invasion, and multidrug resistance protein expression in recipient GBM cells. (**A**,**B**) Representative immunofluorescence images and quantification of Ki67 (**A**) and P-gp (**B**) in U343 cells treated with the indicated exosomes. Nuclei were counterstained with DAPI. (**C**) EdU incorporation assays monitoring the proliferative capacity of U251 cells incubated with the indicated exosomes. (**D**) Representative images and statistical quantification of Transwell migration and invasion assays in U251 cells treated with the indicated exosomes. Panels (**A**–**D**) * vs. PBS group; * vs. (resistant cells + shRNA-NC)-derived exosomes group.

**Figure 5:**
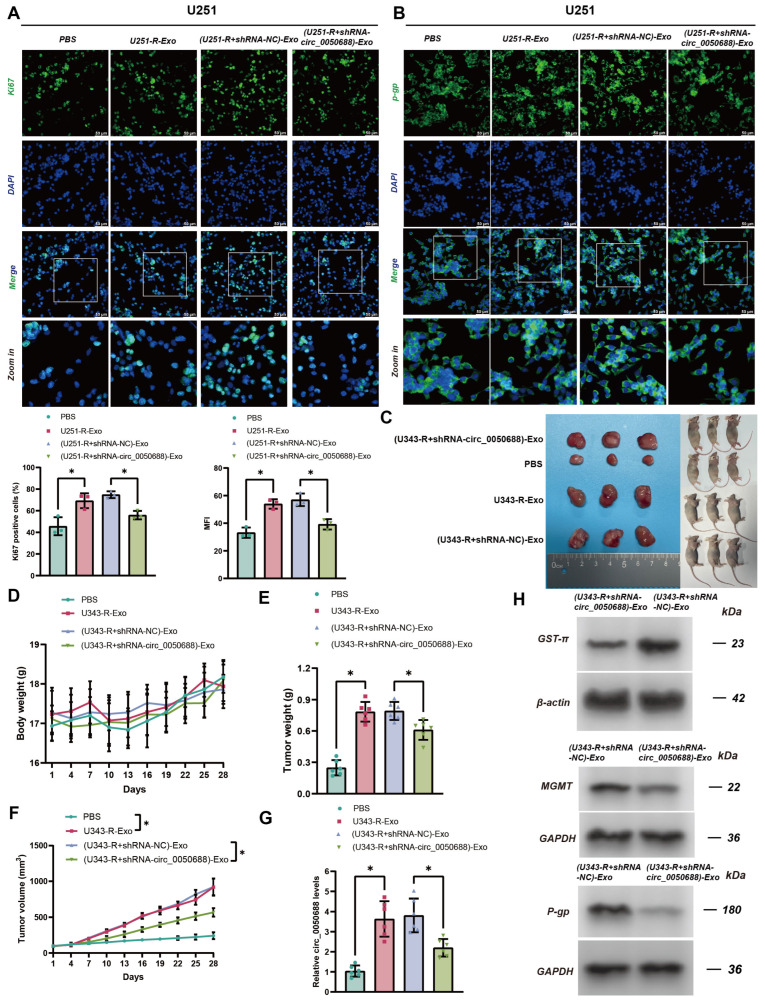
Exosomal circ_0050688 drives TMZ resistance and malignant progression of GBM both in vitro and in vivo. (**A**,**B**) Representative immunofluorescence images and quantification of Ki67 (**A**) and P-gp (**B**) in U251 cells treated with the indicated exosomes. Nuclei were counterstained with DAPI. (**C**) Representative images of excised xenograft tumors and nude mice bearing tumors from the indicated groups. (**D**) Routine monitoring of murine body weights throughout the 28-day experimental period. (**E**,**F**) Final tumor weights (**E**) and tumor growth curves tracking tumor volumes (**F**) across the diverse treatment groups. (**G**) qRT-PCR validation of circ_0050688 expression levels in the excised xenograft tumor tissues. (**H**) Western blot analysis evaluating the protein expression of chemoresistance markers (GST-π, MGMT, and P-gp) in the xenograft tumors. β-actin and GAPDH served as internal loading controls. Panels (**A**,**B**,**E**–**G**) * vs. PBS group; * vs. (resistant cells + shRNA-NC)-derived exosomes group.

To directly evaluate the oncogenic function of exosome-delivered circ_0050688, sensitive cells were treated using exosomes derived from U343-R/U251-R cells modified with shRNA-NC or shRNA-circ_0050688. qRT-PCR verified that the exosomal delivery of circ_0050688 into sensitive cells was significantly impeded when utilizing circ_0050688-depleted exosomes (Figure 3H). Functionally, CCK-8 assays demonstrated that while resistant exosomes (shRNA-NC-Exo) conferred robust TMZ resistance to sensitive cells, this acquired chemoresistance was drastically abolished by shRNA-circ_0050688-Exo (Figure 3I). Moreover, the enhanced cellular proliferation, as vividly evidenced by EdU incorporation (Figure 3J and Figure 4C) and Ki67 immunofluorescence (Figure 4A and Figure 5A), was attenuated when exosomal circ_0050688 was knocked down.

Similarly, Transwell assays indicated that the enhanced aggressive phenotype conferred by resistant exosomes was significantly compromised upon exosomal circ_0050688 silencing in U343 cells (Figure 3K) and U251 (Figure 4D) cells. To further validate these observed phenotypes, immunofluorescence staining confirmed that the exosome-mediated upregulation of the P-gp in sensitive cells was reversed by the depletion of exosomal circ_0050688 (Figure 4B and Figure 5B). Overall, these data demonstrate the exosomal encapsulation of circ_0050688 and its transmission to sensitive cells, thereby disseminating TMZ resistance and augmenting malignant progression via intercellular communication.

### 3.4. Exosome-Mediated Delivery of circ_0050688 Promotes GBM Tumor Growth and Chemoresistance In Vivo

A subcutaneous xenograft model was subsequently developed to evaluate the in vivo capacity of exosomal circ_0050688 to accelerate tumor growth and confer TMZ resistance, thereby validating our cell-based observations. Compared to the PBS control group, administration of exosomes derived from TMZ-resistant cells (U343-R-Exo) or the negative control group dramatically accelerated tumor growth. Crucially, this exosome-mediated oncogenic effect was significantly abrogated when the delivery of exosomes from circ_0050688-deficient resistant cells was performed, resulting in inhibited tumor progression (Figure 5C,E,F). No notable decline in body mass was detected among any of the treatment cohorts. These data suggest that the exosome-mediated delivery system and treatments were well-tolerated (Figure 5D).

Lastly, to probe the molecular basis, the levels of circ_0050688 and representative resistance markers were assessed within the resected tumor specimens. qRT-PCR revealed increased circ_0050688 expression within xenografts subjected to U343-R-Exo; this upregulation was effectively abolished in the (U343-R + shRNA-circ_0050688)-Exo group, verifying that the target transcript was efficiently transported within the animal model (Figure 5G). Consistently, Western blotting indicated a marked upregulation of GST-π, MGMT, and P-gp in the shRNA-NC-Exo cohort. Conversely, these markers were substantially suppressed following treatment with circ_0050688-depleted exosomes (Figure 5H). Collectively, these in vivo findings demonstrate that exosomal circ_0050688 disseminates TMZ resistance and accelerates GBM progression by upregulating essential proteins linked to drug insensitivity.

### 3.5. Identification of circ_0050688 as a Molecular Decoy for miR-508-5p

**Figure 6:**
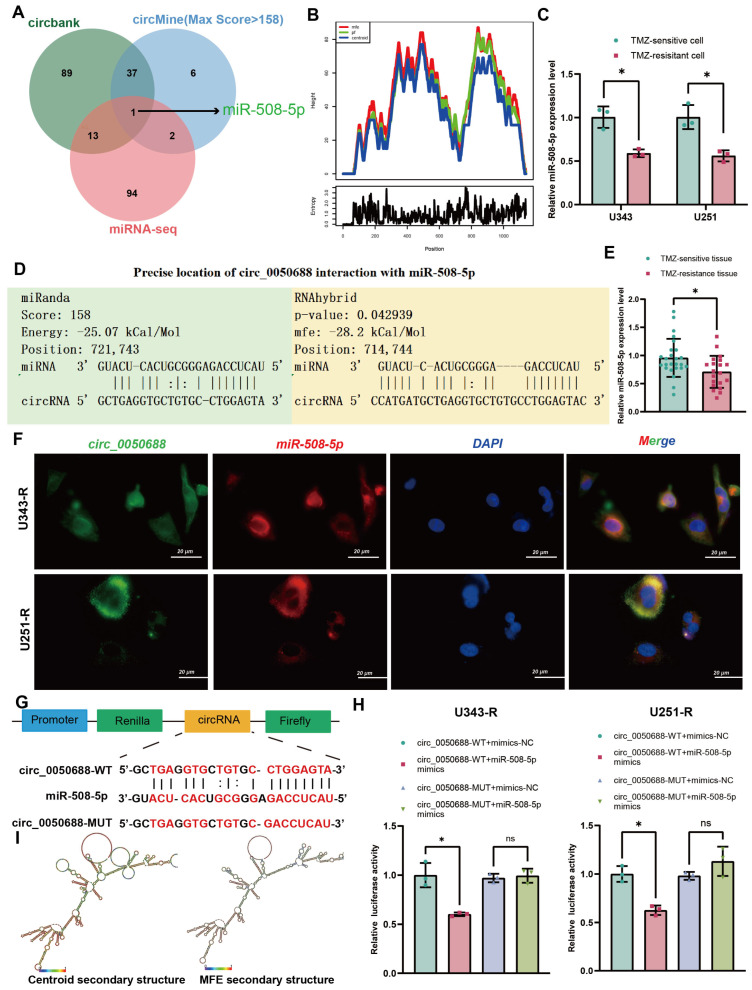
circ_0050688 directly interacts with and acts as a molecular sponge for miR-508-5p. (**A**) A Venn diagram illustrating the intersection of potential target miRNAs predicted by circBank, circMine (Max Score > 158), and differential miRNA sequencing data. (**B**) Positional entropy and minimum free energy (MFE) plot of the circ_0050688 sequence generated via the RNAfold WebServer. (**C**) qRT-PCR analysis of miR-508-5p expression levels in TMZ-sensitive and TMZ-resistant GBM cell lines. (**D**) Bioinformatics prediction of the specific binding interfaces between circ_0050688 and miR-508-5p using miRanda and RNAhybrid algorithms. (**E**) qRT-PCR analysis of miR-508-5p expression in TMZ-sensitive and TMZ-resistant GBM tissues. (*n* = 25 for TMZ-sensitive, *n* = 20 for TMZ-resistant). (**F**) Representative RNA FISH images demonstrating the subcellular colocalization of circ_0050688 (green) and miR-508-5p (red) primarily in the cytoplasm of U343-R and U251-R cells. Nuclei were counterstained with DAPI (blue). Scale bars = 20 μm. (**G**) Schematic of the predicted miR-508-5p binding sites within wild type (WT) circ_0050688 and the designed mutant (MUT) sequence. (**H**) Relative luciferase activity in U343-R and U251-R cells co-transfected with the WT or MUT reporter vectors and miR-508-5p mimics or mimics-NC. (**I**) Predicted centroid and MFE secondary structures of the circ_0050688 transcript. Panel (**C**) * vs. TMZ-sensitive cell group. Panel (**E**) * vs. TMZ-sensitive tissue group. Panel (**H**) * vs. circ_0050688-WT + mimics-NC group; not significant (n.s.) vs. circ_0050688-MUT + mimics-NC group.

Given the predominant cytoplasmic distribution and structural stability of circ_0050688, we hypothesized that it exerts its oncogenic functions via a ceRNA network. To identify potential miRNA targets, we intersected bioinformatics predictions from circBank and circMine (Max Score > 158) with our differential miRNA sequencing data, which pinpointed miR-508-5p as the sole candidate (Figure 6A). To evaluate the thermodynamic feasibility of this interaction, we analyzed the sequence via the RNAfold WebServer. The analysis generated a highly stable minimum free energy (MFE) structure and a consistent centroid secondary structure, confirming the internal backbone of circ_0050688 (Figure 6I). Crucially, mapping the positional entropy alongside the MFE plot revealed that the precise binding interfaces for miR-508-5p (predicted by miRanda and RNAhybrid algorithms) are located within accessible, flexible regions of the circular transcript, providing a structural basis for its sponge function (Figure 6B,D).

Clinically and cellularly, qRT-PCR analysis revealed that miR-508-5p expression was downregulated in both TMZ-resistant GBM cell lines and clinical tissue specimens compared to their sensitive counterparts, exhibiting an inverse expression pattern to that of circ_0050688 (Figure 6C,E). Subcellular colocalization of circ_0050688 and miR-508-5p predominantly within the cytoplasm was further confirmed by RNA FISH (Figure 6F). Finally, dual-luciferase reporter assays provided direct physical evidence of this interaction: a significant reduction in luciferase signal from the wild-type circ_0050688 (circ_0050688-WT) construct was observed upon miR-508-5p overexpression, whereas the mutant reporter (circ_0050688-MUT) with disrupted binding sites remained unaffected (Figure 6G,H). Overall, these data provide evidence for the physical binding of circ_0050688 to miR-508-5p in the GBM cell environment.

### 3.6. circ_0050688 Drives GBM Malignant Progression and TMZ Resistance by Sponging miR-508-5p In Vitro

**Figure 7:**
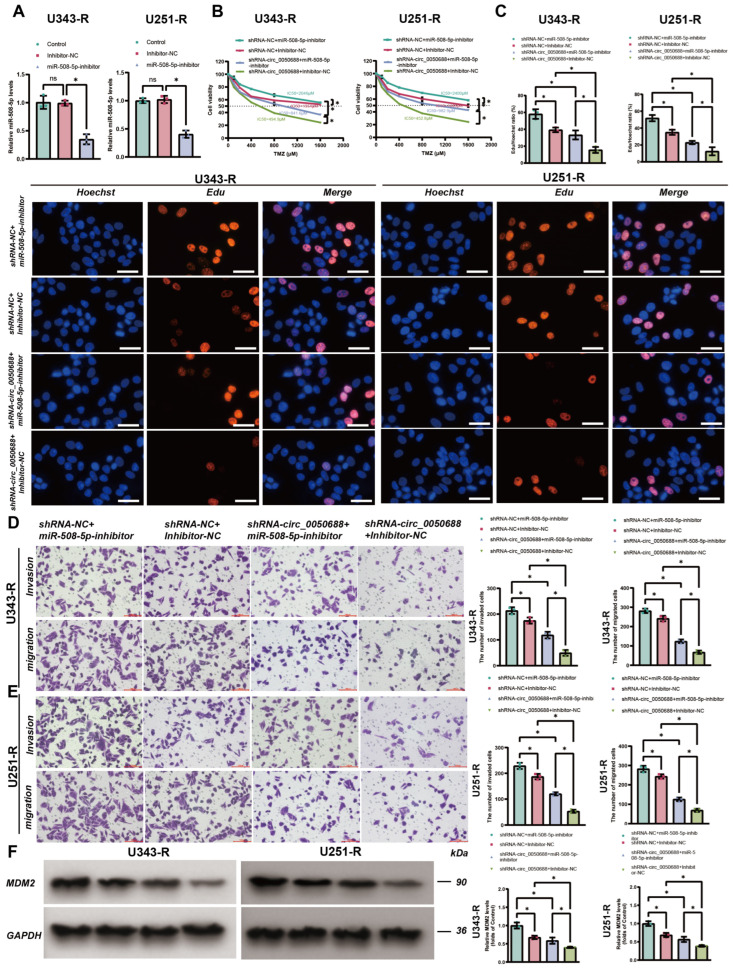
circ_0050688 drives GBM malignant progression and TMZ resistance by sponging miR-508-5p. (**A**) qRT-PCR analysis of miR-508-5p expression in U343-R and U251-R cells transfected with miR-508-5p or control inhibitors. (**B**) Cell viability curves and corresponding TMZ IC_50_ values after indicated co-transfection. IC_50_ was calculated by nonlinear regression (variable-slope dose–response model). (**C**) Representative images and statistical quantification of EdU incorporation assays assessing cell proliferation in the indicated co-transfection groups. Scale bars = 50 μm. (**D**,**E**) Representative images and quantitative analysis of Transwell migration and invasion assays in U343-R (**D**) and U251-R (**E**) cells across the indicated co-transfection groups. Scale bars = 100 μm. (**F**) Western blot analysis of MDM2 in U343-R and U251-R cells following the indicated treatments. GAPDH was used as a loading control. Panel (**A**) not significant (n.s.) vs. Control group; * vs. Inhibitor-NC group. Panel (**B**) * for all bracketed pairwise comparisons. Panels (**C**–**F**) * for pairwise comparisons among experimental groups.

To determine whether circ_0050688 promotes TMZ tolerance and aggressive phenotypes via the sequestration of miR-508-5p, functional restoration assays were conducted. The effective suppression of miR-508-5p by specific inhibitors was first validated (Figure 7A). CCK-8 assays revealed that while circ_0050688 silencing resensitized resistant cells to TMZ (decreasing IC_50_ values) and suppressed cell viability, these effects were reversed by co-transfection with miR-508-5p inhibitors (Figure 7B). Similarly, the impaired proliferation induced by circ_0050688 knockdown, as assessed by EdU assays, was counteracted by miR-508-5p inhibition (Figure 7C).

Furthermore, Transwell assays demonstrated that the impaired motility and invasiveness of circ_0050688-silenced cells were restored via miR-508-5p inhibition (Figure 7D,E). At the molecular level, Western blot analysis confirmed that the expression of MDM2, a key downstream target protein, was significantly suppressed following circ_0050688 silencing, but this effect was rescued by the miR-508-5p inhibitor (Figure 7F). Together, these rescue assays demonstrate that circ_0050688 drives TMZ resistance and GBM progression by sponging miR-508-5p.

### 3.7. Identification of MDM2 as the Direct Functional Target of the circ_0050688/miR-508-5p Axis

**Figure 8:**
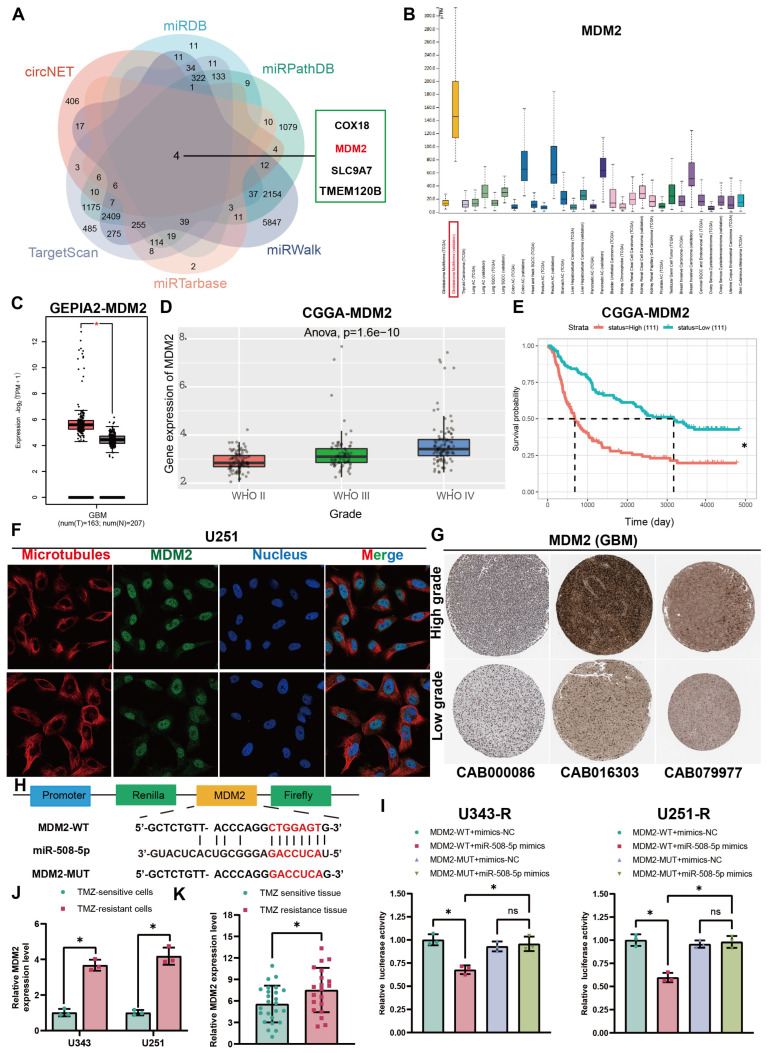
Identification of MDM2 as the direct functional target of the circ_0050688/miR-508-5p axis. (**A**) Venn diagram illustrating the intersection of potential miR-508-5p target genes predicted by six independent bioinformatic databases. (**B**) Pan-cancer expression profile of MDM2 derived from the Human Protein Atlas (HPA). (**C**) Box plots detailing MDM2 mRNA expression levels in GBM tissues compared to normal tissues, sourced from the GEPIA2 database. (**D**) Correlation between MDM2 expression and WHO malignancy grades of glioma based on the CGGA database. (**E**) Kaplan–Meier overall survival curves of glioma patients stratified by MDM2 expression (CGGA database, *n* = 222 patients). (**F**) Representative immunofluorescence images from the HPA Subcellular Atlas demonstrating the predominant nuclear localization of MDM2 in U251 cells. Microtubules (red), MDM2 (green), and nuclei (blue, DAPI) are shown. (**G**) Representative clinical immunohistochemistry (IHC) images from the HPA Pathology Atlas showing elevated MDM2 protein levels in high-grade versus low-grade GBM tissues. (**H**) Schematic of the predicted binding interfaces between the 3′ UTR of MDM2 and miR-508-5p, alongside the design of the MUT vector. (**I**) Dual-luciferase reporter assays in U343-R and U251-R cells co-transfected with MDM2-WT or MDM2-MUT vectors and miR-508-5p mimics or mimics-NC. (**J**) qRT-PCR analysis of MDM2 mRNA levels in TMZ-sensitive and TMZ-resistant GBM cell lines. (**K**) Relative MDM2 expression in TMZ-sensitive and TMZ-resistant clinical GBM tissue specimens, evaluated by qRT-PCR. Panel (**C**) * vs. normal tissue group. Panel (**E**) * vs. low MDM2 group. Panel (**I**) * vs. MDM2-WT + mimics-NC group; * vs. MDM2-WT + miR-508-5p mimics group. Panel (**J**) * vs. TMZ resistant GBM cell lines group. Panel (**K**) * vs. TMZ-sensitive tissue group.

To map the downstream oncogenic effectors governed by the circ_0050688/miR-508-5p axis, we intersected prediction data from six independent databases (miRPathDB, miRDB, miRWalk, miRTarbase, circNET, and TargetScan). This rigorous screening identified the canonical oncogene MDM2, distinguished by its E3 ubiquitin ligase activity and involvement in chemoresistance, as the consensus downstream target (Figure 8A). Pan-cancer analysis via the Human Protein Atlas (HPA) revealed that MDM2 is exceptionally highly expressed in GBM (Figure 8B). Further interrogations utilizing the GEPIA2 and CGGA databases demonstrated that MDM2 expression is significantly elevated in GBM compared to normal tissues (Figure 8C), is positively correlated with the WHO malignancy grades (Figure 8D), and serves as a molecular indicator of poor prognosis within the glioma cohort (Figure 8E). At the protein level, immunofluorescence staining and clinical immunohistochemistry (IHC) data from the HPA corroborated that MDM2 is abundantly localized in the nucleus (Figure 8F) and significantly enriched in high-grade GBM tissues relative to low-grade lesions (Figure 8G).

To validate this axis, bioinformatics algorithms were employed to identify the precise complementary binding interfaces between miR-508-5p and the 3′ UTR of MDM2 (Figure 8H). In both cell lines, the direct interaction was further evidenced by dual-luciferase reporter analysis, in which miR-508-5p mimics significantly suppressed the luciferase activity of the MDM2-WT reporter without affecting the MDM2-MUT construct (Figure 8I). Clinically and cellularly, MDM2 expression was substantially upregulated in TMZ-refractory GBM models and clinical tissue specimens relative to sensitive controls (Figure 8J,K). These comprehensive bioinformatic and physical validations firmly establish MDM2 as a direct, clinically relevant target of miR-508-5p in GBM.

### 3.8. circ_0050688 Drives GBM Malignant Progression and TMZ Resistance Through the miR-508-5p/MDM2 Axis

**Figure 9:**
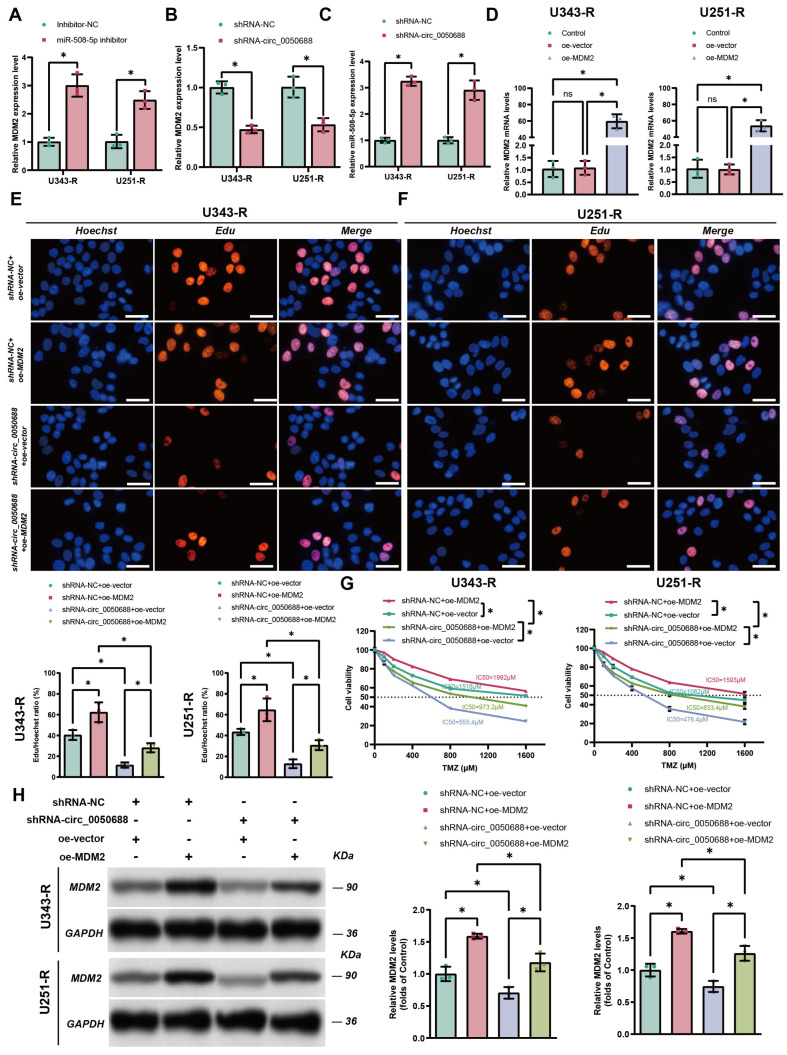
Restoration of MDM2 counteracts the tumor-suppressive and chemo-sensitizing effects of circ_0050688 silencing**.** (**A**) qRT-PCR analysis of MDM2 expression in U343-R and U251-R cells following transfection with a miR-508-5p inhibitor or inhibitor-NC. (**B**,**C**) qRT-PCR validation of MDM2 (**B**) and miR-508-5p (**C**) levels in resistant cells transfected with shRNA-circ_0050688 or shRNA-NC. (**D**) Verification of MDM2 overexpression (oe-MDM2) efficiency at the mRNA level via qRT-PCR. (**E**,**F**) Representative images and statistical quantification of EdU incorporation assays assessing the proliferation of U343-R (**E**) and U251-R (**F**) cells subjected to the indicated rescue co-transfections. Scale bars = 50 μm. (**G**) Cell viability curves and calculated IC_50_ values (via CCK-8 assays) evaluating TMZ sensitivity across the indicated functional rescue groups. (**H**) Western blot analysis evaluating the protein expression of MDM2 in the indicated co-transfection groups. GAPDH served as the internal control. Panel (**A**) * vs. Inhibitor-NC group. Panels (**B**,**C**) * vs. shRNA-NC group. Panel (**D**) not significant (n.s.) vs. Control group; * vs. oe-vector group. Panel (**G**) * for all bracketed pairwise comparisons. Panels (**E**,**F**,**H**) * for pairwise comparisons among experimental groups.

To determine whether circ_0050688 promotes tumorigenesis via the miR-508-5p/MDM2 axis, phenotypic restoration experiments were conducted. miR-508-5p inhibition significantly upregulated MDM2 expression across both resistant models (Figure 9A). Consistently, silencing circ_0050688 alone led to MDM2 downregulation (Figure 9B) and a reciprocal increase in miR-508-5p levels (Figure 9C).

**Figure 10:**
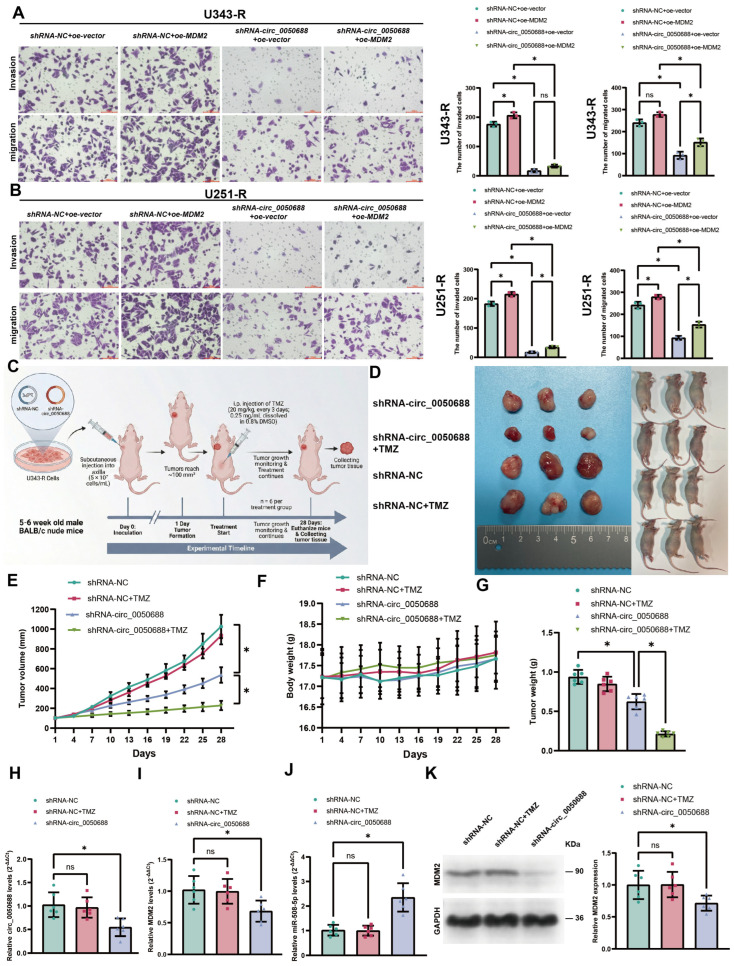
Silencing circ_0050688 restrains xenograft growth and reverses TMZ resistance in vivo. (**A**,**B**) Representative images and quantitative analysis of Transwell invasion and migration assays in U343-R (**A**) and U251-R (**B**) cells across the indicated rescue groups. Scale bars = 100 μm. (**C**) Schematic of the in vivo experimental design for establishing the xenograft model and subsequent TMZ treatment. (**D**) Representative macroscopic images of the excised xenograft tumors and the tumor-bearing nude mice from the respective treatment groups. (**E**) Tumor growth curves tracking tumor volumes across the diverse in vivo treatment groups over the 28-day period. (**F**) Routine monitoring of murine body weights throughout the experimental duration. (**G**) Final weights of the excised xenograft tumors. (**H**–**J**) qRT-PCR validation of circ_0050688 (**H**), MDM2 (**I**), and miR-508-5p (**J**) expression levels in the excised xenograft tumor tissues. (**K**) Western blot analysis confirming the suppression of MDM2 protein expression in vivo following circ_0050688 knockdown. GAPDH served as the internal control. Panels (**A**,**B**) * for pairwise comparisons; not significant (n.s.) for the indicated comparison. Panels (**E**,**G**) * for all bracketed pairwise comparisons. Panels (**H**–**K**) not significant (n.s.) between shRNA-NC and shRNA-NC + TMZ; * vs. shRNA-NC group.

Next, an MDM2 overexpression vector (oe-MDM2) was introduced into circ_0050688-depleted cells, with upregulation confirmed via qRT-PCR (Figure 9D). The impaired replicative capacity following circ_0050688 depletion was largely rescued by MDM2 upregulation, as evidenced by EdU assays (Figure 9E,F). CCK-8 assays revealed that while circ_0050688 silencing re-sensitized the resistant cells to TMZ (indicated by sharp declines in IC50 values), concurrent MDM2 overexpression effectively restored their chemoresistant capacity (Figure 9G). Similarly, ectopic MDM2 expression successfully mitigated the suppression of cell motility resulting from circ_0050688 deficiency (Figure 10A,B). Western blotting confirmed that the MDM2 protein depletion induced by circ_0050688 silencing was rescued by oe-MDM2 (Figure 9H). Collectively, the circ_0050688/miR-508-5p/MDM2 signaling module underpins the aggressive phenotype and chemoresistance of GBM.

### 3.9. Silencing circ_0050688 Restrains Xenograft Growth and Reverses TMZ Resistance In Vivo

Subcutaneous xenografting was performed by injecting U343-R lineages into mice to assess their tumor-forming ability. As illustrated in the experimental paradigm (Figure 10C), once the xenograft tumors were successfully established, mice harboring tumors were randomly allocated into four distinct cohorts (*n* = 6) to evaluate the independent and synergistic effects of circ_0050688 silencing and TMZ administration. Consistent with our in vitro findings, silencing circ_0050688 significantly impaired tumorigenic potential. Strikingly, co-administration of shRNA-circ_0050688 alongside intraperitoneal TMZ injections resulted in a potent synergistic therapeutic effect, resulting in the greatest reduction in both tumor volume (Figure 10D,E) and tumor weight (Figure 10G), without causing notable body weight loss or systemic toxicity (Figure 10F).

To confirm the underlying mechanism in vivo, molecular analyses of excised xenograft tissues were performed. Tumors from the shRNA-circ_0050688 group exhibited reduced circ_0050688 levels (Figure 10H), accompanied by the downregulation of MDM2 mRNA (Figure 10I) and miR-508-5p upregulation (Figure 10J). Western blotting verified that MDM2 protein levels were significantly suppressed following circ_0050688 silencing (Figure 10K). These in vivo data compellingly validate that therapeutic targeting of circ_0050688 overcomes acquired TMZ resistance and restrains GBM progression by disrupting the miR-508-5p/MDM2 signaling axis.

## 4. Discussion

GBM represents the most prevalent and aggressive malignant primary intracranial malignancy worldwide. Notwithstanding conventional interventions consisting of aggressive operative excision followed sequentially by auxiliary chemoradiotherapy, patient survival remains dismally poor. Correspondingly, acquired resistance to TMZ remains one of the main obstacles in GBM management. Accordingly, deciphering the underlying pathways that govern TMZ refractoriness, alongside identifying reliable predictive biomarkers and actionable therapeutic targets, is of paramount clinical importance. Here, utilizing circRNA microarray profiling, we identify circ_0050688 as a novel, markedly upregulated transcript in GBM. In vitro assays demonstrate that circ_0050688 not only confers TMZ resistance but also facilitates the expansion and motility of GBM cells, underscoring its pivotal contribution to malignant advancement. These phenotypic changes are underpinned by a circ_0050688/miR-508-5p/MDM2-driven ceRNA mechanism. More importantly, high expression of circ_0050688 is an independent poor prognostic factor for GBM patients receiving standard TMZ treatment. Importantly, we establish that circ_0050688 is highly enriched in and horizontally transmitted via exosomes. These vesicles are internalized by sensitive cells, leading to the delivery of circ_0050688 and subsequent induction of chemoresistance in the recipient population. Blocking exosomal communication effectively impairs this intercellular transfer and reverses the resistant phenotype. Furthermore, in vivo xenograft studies confirm that circ_0050688 silencing synergistically enhances the therapeutic efficacy of TMZ, leading to a marked reduction in tumor burden. Collectively, our findings provide a compelling mechanistic rationale and highlight a targetable vulnerability to address chemoresistance in GBM.

Well-documented as a vital anti-oncogenic factor and a central regulator of multidrug resistance, miR-508-5p orchestrates essential oncogenic processes. Accumulating evidence indicates that miR-508-5p is frequently downregulated across diverse malignancies, including lung adenocarcinoma [38] and hepatocellular carcinoma [39], where it exerts tumor-suppressive functions. Notably, previous reports have delineated its role in governing multidrug resistance in gastric cancer via the targeted repression of ABCB1 and ZNRD1 [40]. Furthermore, diminished miR-508-5p expression has been observed in patients with relapsed acute lymphoblastic leukemia, establishing it as a putative biomarker for therapeutic resistance [41]. Within the context of GBM, ectopic miR-508-5p has been proven to impair cell viability; however, its precise mechanistic contributions and the broader regulatory networks remain to be fully elucidated [42]. Our results reveal that miR-508-5p expression is markedly diminished in GBM cells characterized by TMZ resistance. The physical association between circ_0050688 and miR-508-5p within the cytoplasm was substantiated by dual-luciferase reporter and FISH co-localization analyses. Functional validation further demonstrated that circ_0050688 antagonizes the tumor-suppressive effects of miR-508-5p, thereby promoting cell proliferation and TMZ resistance.

It is noteworthy that the TMZ-resistant microenvironment is shaped by a multitude of dynamic factors, including diverse ncRNA networks. For instance, while our previous work demonstrated that exosomal circ_0017636 is downregulated in refractory GBM [23], the current study identifies circ_0050688 as a markedly upregulated oncogenic driver. We hypothesize that rather than forming a direct inverse regulatory loop, these contrasting circRNAs act as distinct nodes within parallel resistance pathways. Their divergent expression trajectories likely reflect the profound spatiotemporal heterogeneity of the tumor microenvironment, where different circRNAs are orchestrated by distinct upstream stimuli—such as hypoxia, metabolic stress, or specific transcription factor activation—during prolonged TMZ exposure. Fully mapping this multi-target circRNA interactome remains an essential future direction for optimizing targeted RNA-directed combinatorial therapeutic strategies.

MDM2 mediates the degradation of the tumor suppressor p53 and is critically involved in cancer progression, metastasis, and chemoresistance [43]. Across various malignancies, aberrant MDM2 levels correlate with poor prognosis and therapy resistance [44,45,46]. However, its role in modulating TMZ sensitivity in GBM, along with its upstream regulatory network, remains largely elusive. Our study identifies the circ_0050688/miR-508-5p/MDM2 axis as a central signaling cascade driving GBM chemoresistance. By acting as a ceRNA, circ_0050688 effectively sequesters miR-508-5p, thereby preventing the post-transcriptional silencing of MDM2. Silencing circ_0050688 disrupts this regulatory loop, suppressing MDM2 protein levels and consequently restoring TMZ sensitivity. This mechanism provides a clear molecular rationale for why MDM2 is persistently upregulated in resistant GBM and offers a promising target for therapeutic intervention.

Clarifying the circ_0050688/miR-508-5p/MDM2 signaling module explains the molecular basis of chemoresistance while unmasking an actionable vulnerability for integrated therapeutic strategies. Silencing circ_0050688 suppresses MDM2, thereby stabilizing p53 and lowering the apoptotic threshold for radiotherapy sensitization [47,48,49]. Beyond direct cytotoxicity, the MDM2-p53 network serves as a key orchestrator of the immune microenvironment. Inhibiting this axis may attenuate immune checkpoints like PD-L1 and promote cytotoxic T-lymphocyte recruitment, potentially augmenting immune checkpoint blockade [50,51]. Therefore, integrating circ_0050688-targeted strategies could potently augment the Stupp protocol and emerging multimodal treatments.

Furthermore, the dynamic evolution of the tumor microenvironment is fundamentally driven by vesicular communication [52,53,54]. Glioblastoma-derived exosomes function as essential intercellular vehicles, disseminating bioactive non-coding RNAs to adjacent or distant niches. By exacerbating intratumoral heterogeneity and promoting disease progression, this exosome-mediated network ultimately propagates the TMZ-resistant phenotype throughout the TME [55,56]. We demonstrate that circ_0050688 is encapsulated within exosomes and shuttled to TMZ-sensitive recipient cells, where it activates the miR-508-5p/MDM2 axis to confer acquired resistance. These findings suggest two major clinical applications. First, given its stability in biofluids, exosomal circ_0050688 represents a potential non-invasive liquid biopsy biomarker. Monitoring its levels in serum or cerebrospinal fluid could enable real-time tracking of TMZ resistance and personalized treatment adjustment. Second, targeting the exosomal circ_0050688/MDM2 axis is a viable therapeutic strategy. Combining circ_0050688-targeting antisense oligonucleotides or siRNAs with MDM2 inhibitors and standard TMZ could intercept the intercellular dissemination of resistance, re-sensitize GBM cells, and improve outcomes for recurrent or refractory cases. Overall, targeting this exosomal network offers a feasible translational approach to overcome GBM chemoresistance.

The acquisition of TMZ resistance in GBM involves complex and compensatory signaling pathways. Our findings indicate that circ_0050688 (also annotated as circWDR62) contributes to this process through a parallel resistance mechanism. Previously, we demonstrated that intracellular circ_0050688 promotes DNA repair by regulating the miR-370-3p/MGMT axis [21]. Expanding upon those findings, the current study shows that exosomal transfer of circ_0050688 additionally activates the miR-508-5p/MDM2 cascade. Therefore, we propose a “dual-hit” model for circ_0050688-mediated resistance. While the circ_0050688/MGMT axis functions in a cell-autonomous manner to repair TMZ-induced DNA damage, the exosome-mediated circ_0050688/MDM2 axis operates intercellularly to induce P-gp-mediated multidrug efflux in recipient cells. Together, these intracellular and microenvironmental pathways operate synergistically to maintain TMZ refractoriness, highlighting this circular RNA as a viable therapeutic target.

Nevertheless, several limitations in the present study should be acknowledged. Several limitations warrant consideration. Although our current cohort establishes a foundational link between circ_0050688 and TMZ resistance, the restricted number of refractory clinical specimens necessitates future validation across extensive, multicenter prospective populations to solidify its clinical utility. Furthermore, while the subcutaneous xenograft system successfully yielded proof-of-principle efficacy data, we acknowledge its inherent limitations. This model was utilized in the initial phase of our study as it allows for precise, real-time monitoring of tumor volume kinetics, providing a simplified in vivo baseline to establish the exosome-mediated ceRNA network effect. However, it admittedly lacks the physiological complexity of the central nervous system, specifically the BBB and dynamic glial interactions. Therefore, subsequent orthotopic investigations are imperative to accurately map the spatial spreading of resistance within the authentic intracranial microenvironment. Furthermore, building upon these mechanisms, future translational endeavors could explore surface-engineering technologies to maximize active BBB penetration, ensuring precise therapeutic targeting of this circRNA network in GBM [57,58,59,60,61]. Equally importantly, the nude mouse model inherently lacks a functional immune system and native microglial niche. Consequently, we could not evaluate exosomal circ_0050688’s impact on broader TME reprogramming, such as macrophage interactions. Future studies utilizing immunocompetent orthotopic models are imperative to fully map this immune-mediated resistance network. Third, while we characterized the circ_0050688/miR-508-5p/MDM2 axis as a cytoplasmic ceRNA network, the precise downstream effector mechanisms of MDM2 warrant deeper consideration in the context of varying genetic backgrounds. Specifically, the GBM cell lines utilized in this study exhibit distinct TP53 statuses: U343 harbors wild-type p53 [62], whereas U251 is well-documented to carry a TP53 mutation [63]. While the canonical MDM2/wild-type p53 degradation axis [43] readily explains the therapy evasion observed in U343 cells, this classic paradigm alone fails to account for the identical chemoresistant phenotypes robustly acquired by the p53-mutant U251 line. This dichotomy suggests that MDM2 concurrently functions through mutant p53-dependent interactions or p53-independent pathways. As supported by emerging literature, cytoplasmic MDM2 can directly orchestrate aggressive phenotypes, such as the epithelial–mesenchymal transition, and drive multidrug resistance independently of its nuclear role [64]. Crucially, this p53-independent regulatory capacity perfectly aligns with our experimental observations, where exosomal circ_0050688 transfer and subsequent MDM2 elevation led to a profound phenotypic induction of the multidrug resistance protein P-gp in both WT and mutant cell lines. Although the exact interactome of this MDM2/P-gp signaling cascade requires further mapping, our findings emphasize that MDM2 universally reshapes the chemoresistant microenvironment by leveraging both p53-dependent and -independent axes [65,66].

Beyond experimental limitations, clinical translation of exosome-mediated circ_0050688 interventions faces several technical hurdles. First, the regulatory networks that dictate the preferential sorting of circ_0050688 into exosomal vesicles require further clarification. Identifying molecular shuttles, such as hnRNPA2B1 or specific sequence motifs, is required to develop controlled loading technologies for consistent therapeutic dosing [67]. Second, standardized production of clinical-grade exosomes remains challenging. Although the lack of free 5′ or 3′ termini in circRNAs protects them from being degraded by exonucleases [68], achieving superior purity and manufacturing reliability in compliance with GMP standards is fundamental. Advanced manufacturing platforms, such as hollow-fiber bioreactors and tangential flow filtration, may be required to scale production [69,70]. Lastly, leveraging patient-specific or MSC-sourced exosomal vesicles represents a viable strategy to mitigate host immune responses, facilitating the development of safe and effective targeted therapies [71,72].

Altogether, our findings identify circ_0050688 as an upregulated oncogenic transcript in TMZ-resistant GBM. Functionally, it drives cellular proliferation, migration, invasion, and chemoresistance in vitro. Mechanistically, circ_0050688 orchestrates these malignant phenotypes by sequestering miR-508-5p, then sustaining MDM2 levels. Notably, our results indicate that the vesicular-shuttled circ_0050688 effectively disseminates TMZ-refractory traits to formerly drug-responsive GBM populations. Collectively, the elucidation of the exosomal circ_0050688/miR-508-5p/MDM2 signaling axis provides fundamental molecular insights into GBM therapeutic evasion. While further investigations are warranted to evaluate its applicability across other malignancies, our findings establish exosomal circ_0050688 as a promising avenue for overcoming chemoresistance in the GBM therapeutic landscape.

## 5. Conclusions

Collectively, this work highlights the overexpression of circ_0050688 in GBM models exhibiting TMZ insensitivity. Current findings define a molecular axis in which circ_0050688 drives GBM progression and chemoresistance through competitive miR-508-5p binding, consequently augmenting MDM2 levels. Moreover, circ_0050688 packaging into exosomes disseminates resistance to sensitive recipients, highlighting its contribution to the niche-mediated signaling network. Taken together, our observations provide a comprehensive framework for understanding the molecular drivers of GBM chemoresistance and suggest that targeting the exosomal circ_0050688/miR-508-5p/MDM2 axis represents a therapeutic strategy to resensitize tumors and improve clinical outcomes.

## Data Availability

Information supporting the current results can be obtained from the corresponding authors following a legitimate request.

## Supplementary Materials

The following supporting information can be downloaded at https://www.mdpi.com/article/10.3390/biom16060906/s1, Figure S1: Forest plot of multivariate Cox regression analysis for overall survival. Multivariate Cox regression was performed to evaluate the independent prognostic value of circ_0050688 in GBM patients receiving TMZ treatment, adjusting for age and MGMT promoter methylation status. Squares represent hazard ratios (HR), horizontal lines indicate 95% confidence intervals (CI), and the vertical dashed line marks the null value of HR = 1; Table S1: Primers used in qRT-PCR; Table S2: The specific sequences of oligonucleotides. File S1: Original images of Western Blot.

## Abbreviations

CCK-8	Cell Counting Kit-8
ceRNA	Competing endogenous RNA
CGGA	Chinese Glioma Genome Atlas
CircRNAs	Circular RNAs
FISH	Fluorescence in situ hybridization
GBM	Glioblastoma
GC	Gastric cancer
gDNA	Genomic DNA
GSC	Glioma stem cell
IC_50_	The maximum half inhibitory concentration
MDM2	Murine double minute-2
MFE	Minimum free energy
NTA	Nanoparticle tracking analysis
qRT-PCR	RNA extraction and quantitative reverse transcription–polymerase reaction
RBP	RNA-binding proteins
shRNA	Short hairpin RNA
TEM	Transmission electron microscopy
TMZ	Temozolomide
