# Supplementary material for: Exosomal circ_0050688 Shapes a Chemoresistant Microenvironment by Driving Spatial Resistance Spreading in Glioblastoma via the MDM2 Pathway
Source: Biomolecules. 2026 Jun 18;16(6):906. doi: 10.3390/biom16060906 (PMC13296696; doi:10.3390/biom16060906)

Figure2F

GST-π (U343-R)

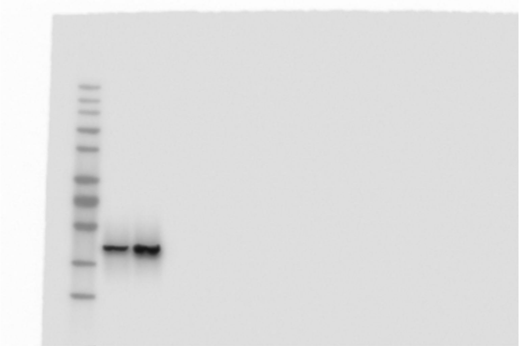

GST-π (U251-R)

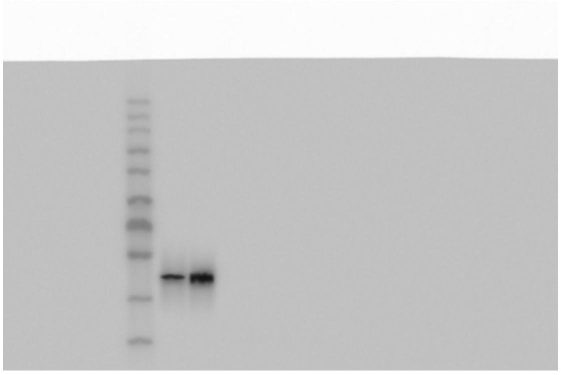

β-actin (U343-R)

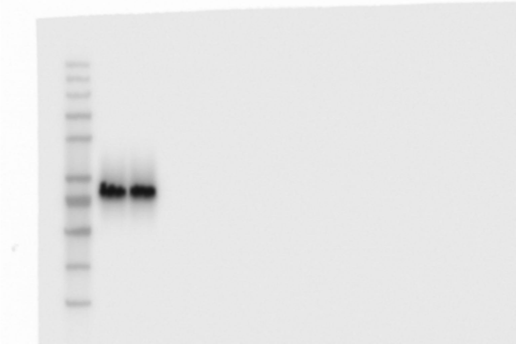

β-actin (U251-R)

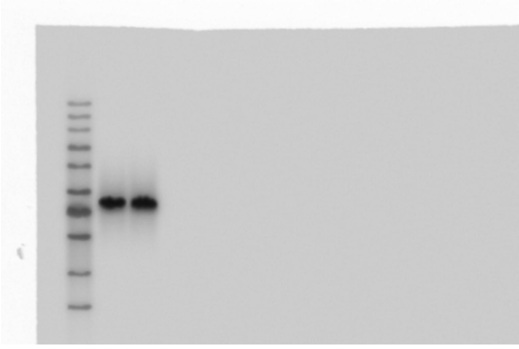

MGMT (U343-R)

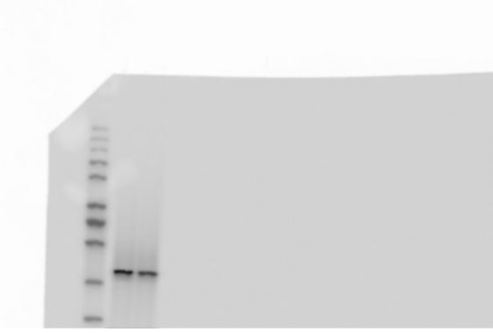

MGMT (U251-R)

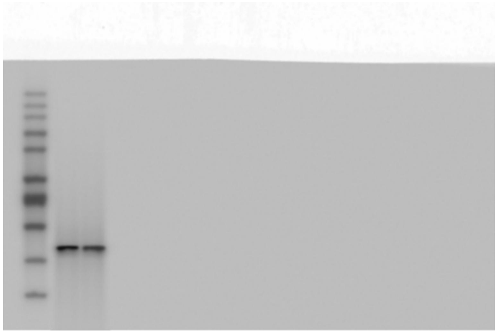

GAPDH (U343-R)

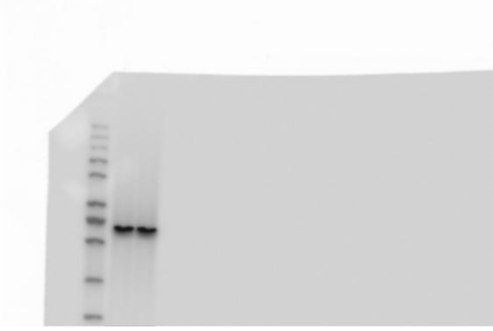

GAPDH (U251-R)

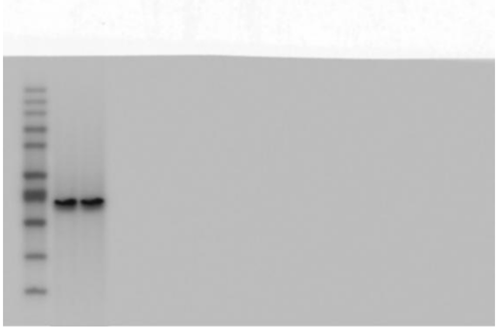

P-gp (U343-R)

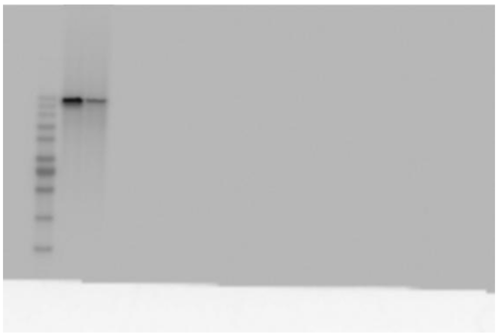

P-gp (U251-R)

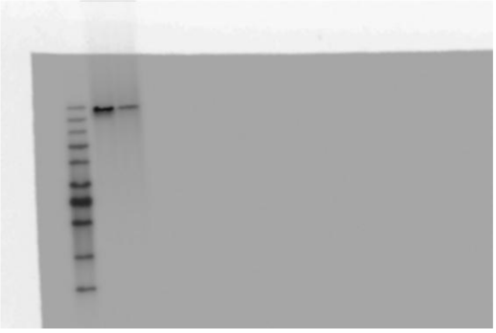

GAPDH (U343-R)

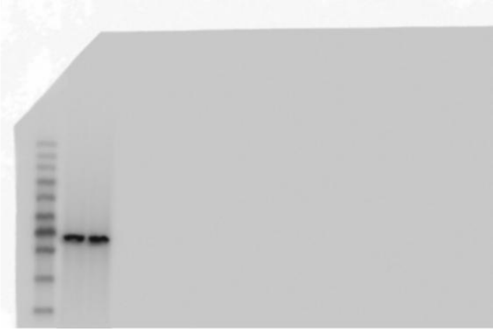

GAPDH (U251-R)

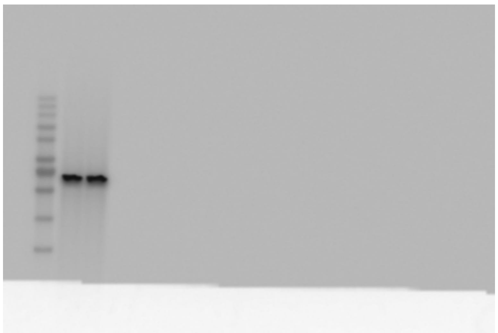

Figure3C

CD63 (U343-Exo U343-R-Exo)

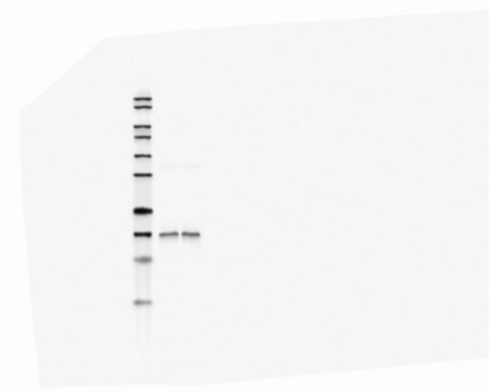

CD63 (U251-Exo U251-R-Exo)

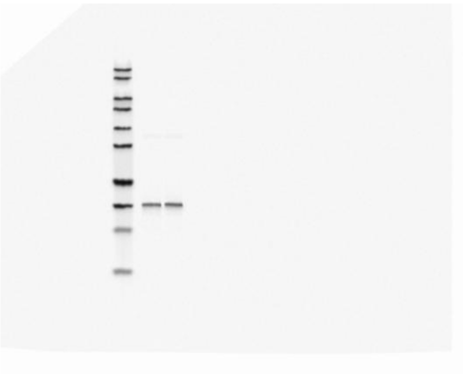

TSG101 (U343-Exo U343-R-Exo)

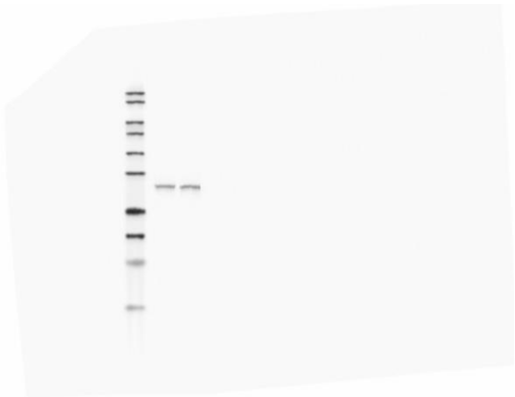

TSG101 (U251-Exo U251-R-Exo)

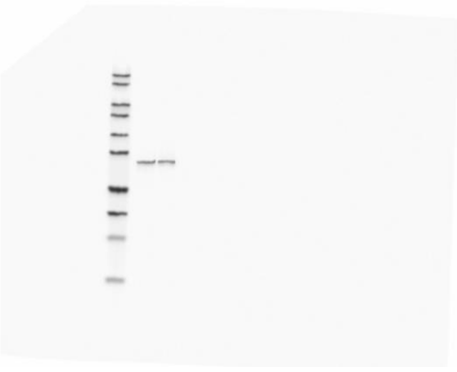

Calnexin (U343-Exo U343-R-Exo)

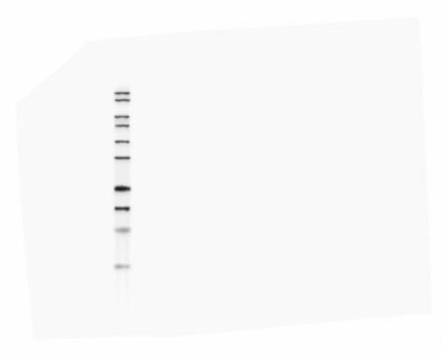

Calnexin (U251-Exo U251-R-Exo)

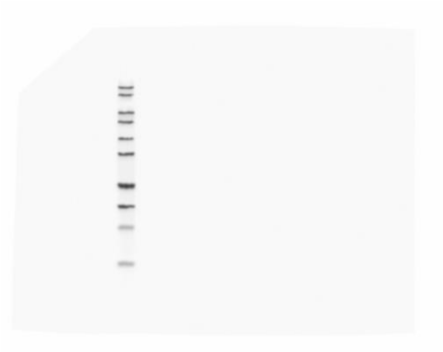

Figure5H

GST-π

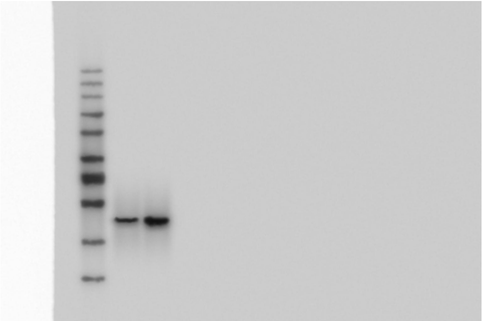

β-actin

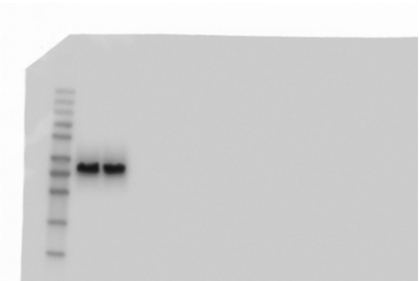

MGMT

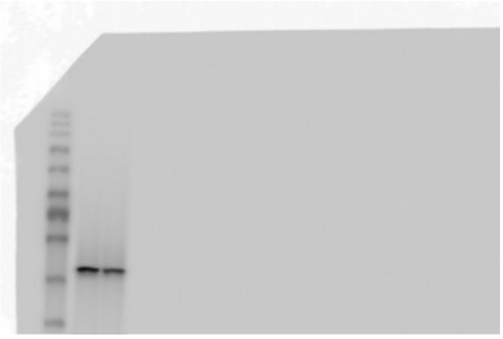

GAPDH

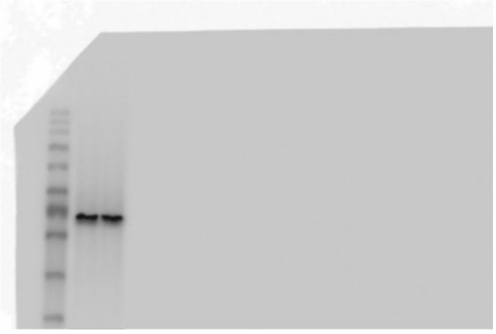

P-gp

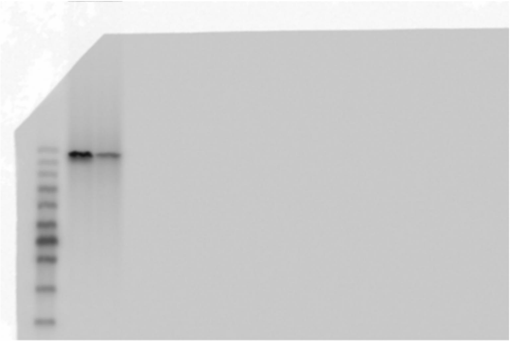

GAPDH

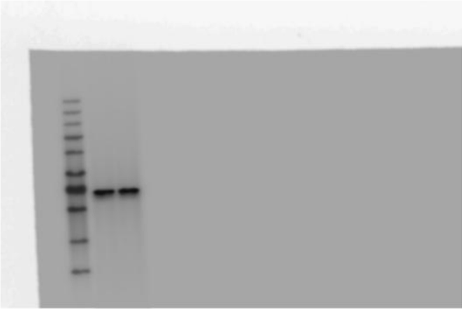

Figure7F

MDM2(U343-R)

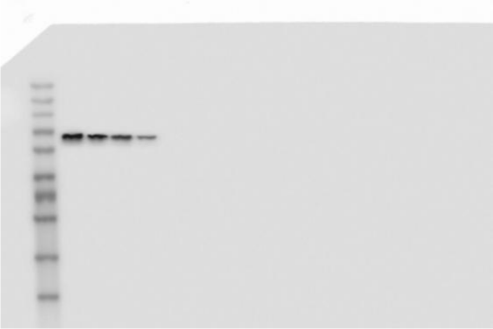

MDM2(U251-R)

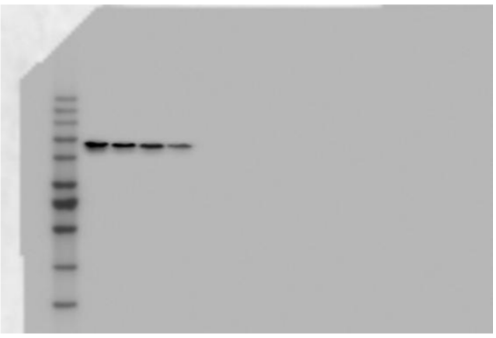

GAPDH (U343-R)

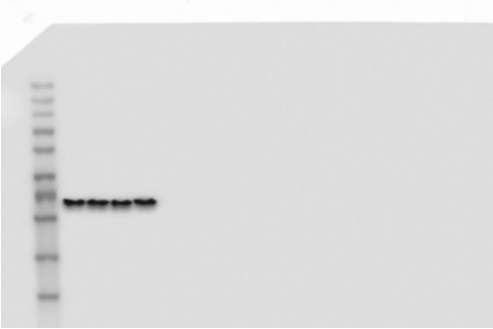

GAPDH (U251-R)

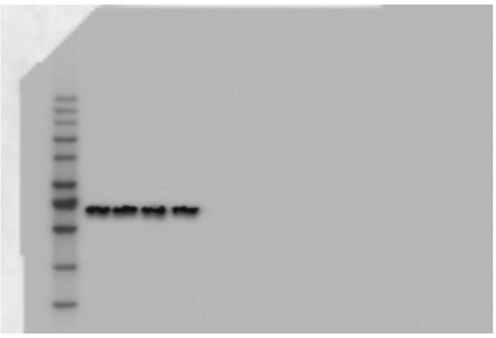

Figure9H

MDM2(U343-R)

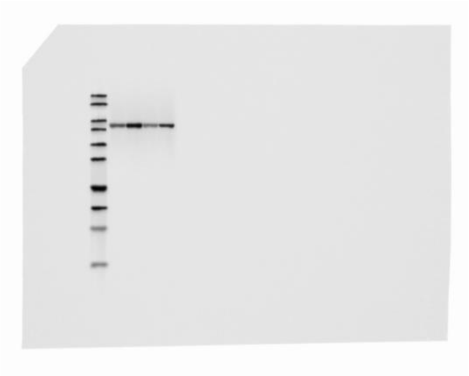

MDM2(U251-R)

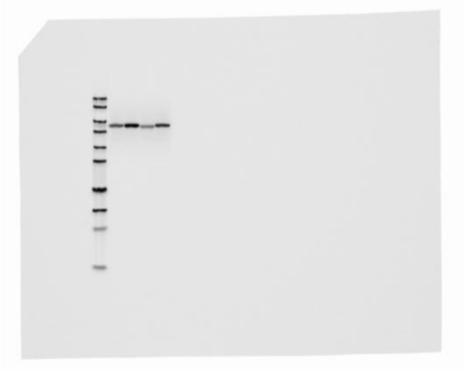

GAPDH (U343-R)

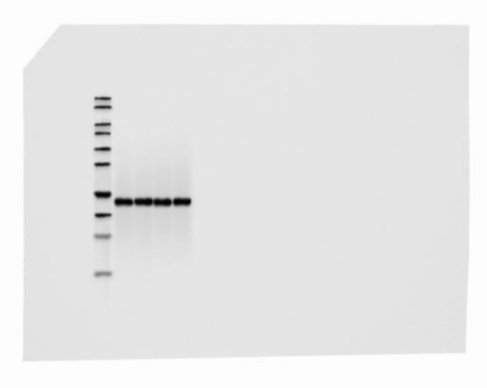

GAPDH (U251-R)

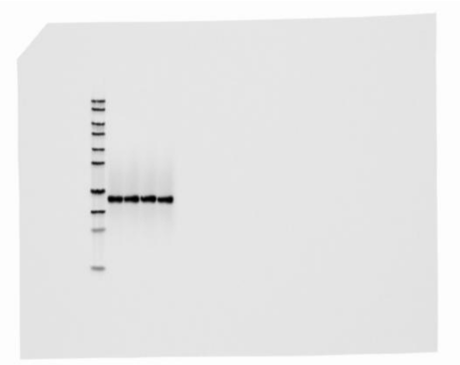

Figure10K

MDM2

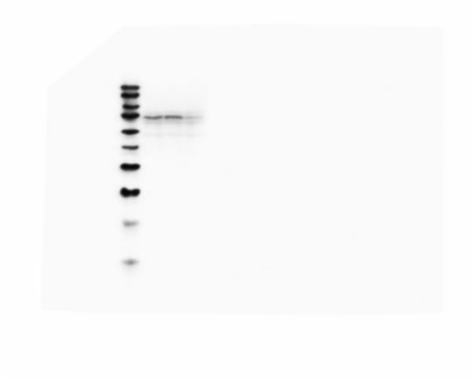

GAPDH

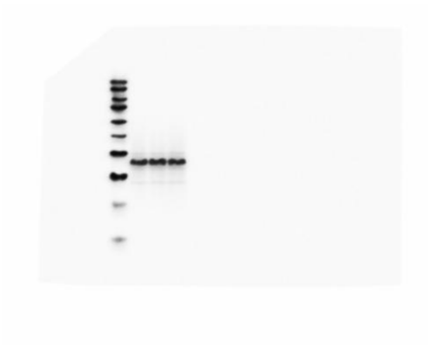

Supplement: Supplementary file 1 [file biomolecules-16-00906-s001.zip › File S1.pdf]
