# Supplementary material for: Exosomal circ_0050688 Shapes a Chemoresistant Microenvironment by Driving Spatial Resistance Spreading in Glioblastoma via the MDM2 Pathway
Source: Biomolecules. 2026 Jun 18;16(6):906. doi: 10.3390/biom16060906 (PMC13296696; doi:10.3390/biom16060906)
Supplement: Supplementary file 1 [file biomolecules-16-00906-s001.zip › Supplementary_Material-TableS1.pdf]

Supplementary Material

Supplementary Table S1

Primers used in qRT-PCR.

Primers		Sequence (5’→3’)
hsa_circ_0050688 (Convergence)	Forward	TGTCAGCCAGGAGCTCATCTTC
	Reverse	TATACACGCAGGACAGCCACTG
hsa_circ_0050688 (Divergent)	Forward	CATCACCCAGAAGTACGTGGC
	Reverse	AGGAAGACAGGGAGACCTCAC
GAPDH (Convergence)	Forward	GGAGCGAGATCCCTCCAAAAT
	Reverse	GGCTGTTGTCATACTTCTCATGG
GAPDH	Forward	GCTGAGTACGTCGTGGAGTC
(Divergent)	Reverse	GAGAACAGTGAGCGCCTAGTG
miR-508-5p	Forward	ACACTCCAGCTGGGTACTCCAGAGGGCGTCACT
	Reverse	TGGTGTCGTGGAGTCG
U6	Forward	CTCGCTTCGGCAGCACA
	Reverse	AACGCTTCACGAATTTGCGT
MDM2	Forward	ACCCTGGTTAGACCAAAGCC
	Reverse	TGGCACGCCAAACAAATCTC
