# Supplementary material for: Exosomal circ_0050688 Shapes a Chemoresistant Microenvironment by Driving Spatial Resistance Spreading in Glioblastoma via the MDM2 Pathway
Source: Biomolecules. 2026 Jun 18;16(6):906. doi: 10.3390/biom16060906 (PMC13296696; doi:10.3390/biom16060906)
Supplement: Supplementary file 1 [file biomolecules-16-00906-s001.zip › Supplementary_Material-TableS2.pdf]

# *Supplementary Material*

**Supplementary Table S2**

The specific sequences of oligonucleotides

| **Name** | **Sequence** |
| --- | --- |
| miR-508-5p inhibitor | Sense: GGCAAAGUUUAAGAUCCUU |
| Inhibitor-NC | Sense: CAGUACUUUUGUGUAGUACAA |
| miR-508-5p mimic | Sense: UACUCCAGAGGGCGUCACUCAUG<br>Antisense: UGAGUGACGCCCUCUGGAGUANN |
| mimic-NC | Sense: UUGUACUACACAAAAGUACUG<br>Antisense: GUACUUUUGUGUAGUACAANN |
| shRNA-NC | CCGGTCGGAACCTGCAGCTTACCGTACGGTAAGCTGCAGGT TCCGTTTTTTG; |
| shRNA-hsa_circ_0050688#1 | CCGGGTGAGGTCTCCCTGTCTTCCTCTCGAGAGGAAGACAG GGAGACCTCACTTTTTG; |
| shRNA-hsa_circ_0050688#2 | CCGGAATGTGAGGTCTCCCTGTCTTCTCGAGAAGACAGGGA GACCTCACATTTTTTTG |
| oe-MDM2 | Forward primer:<br>5’- CCGGTACCATGTGCAATACCAACATGTC- 3’;<br>Reverse primer:<br>5’- GGGGATATCCTAGGGGAAATAAGTTAGCACA- 3’ |
| Fluorescence probe | 5’- FITC- ACAGGGAGACCTCACATTGC- 3’ |
