# Supplementary figures and images for: Exosomal circ_0050688 Shapes a Chemoresistant Microenvironment by Driving Spatial Resistance Spreading in Glioblastoma via the MDM2 Pathway
Source: Biomolecules. 2026 Jun 18;16(6):906. doi: 10.3390/biom16060906 (PMC13296696; doi:10.3390/biom16060906)

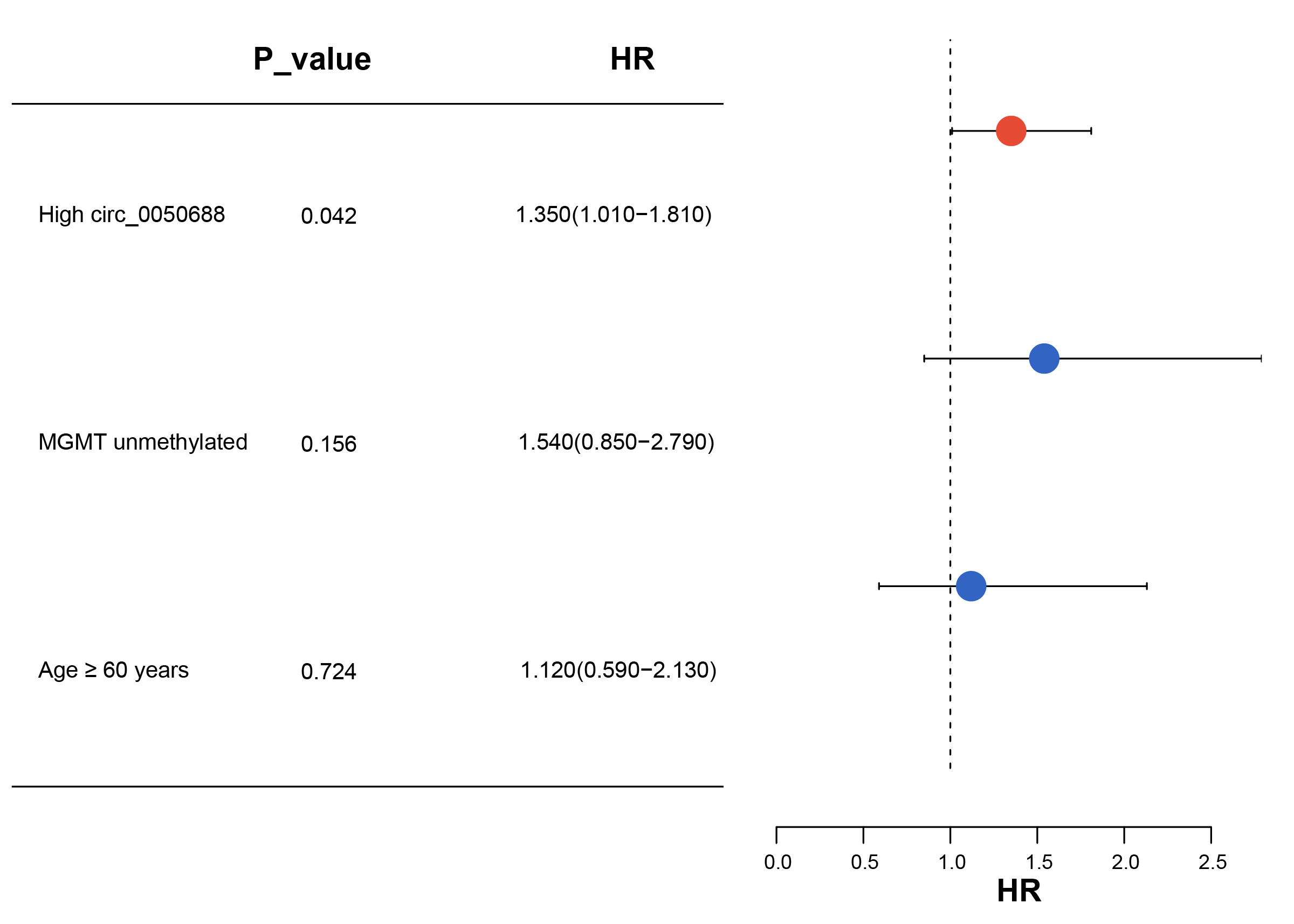

Supplement: Supplementary file 1 [file biomolecules-16-00906-s001.zip › Figure S1.jpg]
